# Genetic diversity, linkage disequilibrium, population structure and construction of a core collection of *Prunus avium* L. landraces and bred cultivars

**DOI:** 10.1186/s12870-016-0712-9

**Published:** 2016-02-24

**Authors:** José Antonio Campoy, Emilie Lerigoleur-Balsemin, Hélène Christmann, Rémi Beauvieux, Nabil Girollet, José Quero-García, Elisabeth Dirlewanger, Teresa Barreneche

**Affiliations:** INRA, UMR 1332 de Biologie du Fruit et Pathologie, F-33140 Villenave d’Ornon, France; University Bordeaux, UMR 1332 de Biologie du Fruit et Pathologie, F-33140 Villenave d’Ornon, France; Current address: CNRS, UMR 5602 GEODE, Géographie de l’environnement, F-31058 Toulouse, France; INRA, UAR 0415 SDAR, Services Déconcentrés d’Appui à la Recherche, F 33140 Villenave d’Ornon, France; Current address: INRA, ISVV, UMR Ecophysiologie et Génomique Fonctionnelle de la Vigne, F 33140 Villenave d’Ornon, France

**Keywords:** Association genetics, Core collection, Discriminant analysis, Genetic diversity, Germplasm management, Linkage disequilibrium, Population structure, *Prunus avium*

## Abstract

**Background:**

Depiction of the genetic diversity, linkage disequilibrium (LD) and population structure is essential for the efficient organization and exploitation of genetic resources. The objectives of this study were to (i) to evaluate the genetic diversity and to detect the patterns of LD, (ii) to estimate the levels of population structure and (iii) to identify a ‘core collection’ suitable for association genetic studies in sweet cherry.

**Results:**

A total of 210 genotypes including modern cultivars and landraces from 16 countries were genotyped using the RosBREED cherry 6 K SNP array v1. Two groups, mainly bred cultivars and landraces, respectively, were first detected using STRUCTURE software and confirmed by Principal Coordinate Analysis (PCoA). Further analyses identified nine subgroups using STRUCTURE and Discriminant Analysis of Principal Components (DAPC). Several sub-groups correspond to different eco-geographic regions of landraces distribution. Linkage disequilibrium was evaluated showing lower values than in peach, the reference *Prunus* species. A ‘core collection’ containing 156 accessions was selected using the maximum length sub tree method.

**Conclusion:**

The present study constitutes the first population genetics analysis in cultivated sweet cherry using a medium-density SNP (single nucleotide polymorphism) marker array. We provided estimations of linkage disequilibrium, genetic structure and the definition of a first INRA’s Sweet Cherry core collection useful for breeding programs, germplasm management and association genetics studies.

**Electronic supplementary material:**

The online version of this article (doi:10.1186/s12870-016-0712-9) contains supplementary material, which is available to authorized users.

## Background

*Prunus avium* L*.* is an economically important temperate species exploited as timber, fruit or rootstock. In Europe, sweet cherry, the cultivated form of *P. avium*, is grown in large areas. Cherries are very appreciated not only for their taste and flavor but because they are the first stone fruits in the markets after the winter. In 2013, Western Europe sweet cherry production represented the 4th one in the world (118,343 tons) according to FAO data (www.fao.org).

*Prunus avium* originated likely in an area between the Black and the Caspian Seas [1, 2]. Stones dated from Neolithic or from Bronze Age found in Central Europe [3] suggested that wild cherry has spread until the extremity of its present area of distribution very early and well before its domestication [4]. Sweet cherry was probably domesticated in the *Prunus avium* area of origin but the hypothesis of several different domestication events from different wild populations cannot be discarded [4]. First cultivated in Greece [5], sweet cherry was later spread all over Europe. Its cultivation seems to be very old, its grafting technique was already described by the Roman writer Varo BC, and Pliny (23–79 AD) gave information of eight distinct cultivars [6, 7]. As a result of centuries of natural and human selection a multitude of cherry landraces were raised in Europe. The economic and social status of cherries has changed in European societies between classical and medieval times [8]. These fruits played an important social role in the medieval elite diet regime [9] before becoming a more common fruit during the later centuries [8, 10].

Although many landraces have been lost, a large diversity still exists in Europe (i.e.: 900 cherry landraces are reported in the European *Prunus* database). On the contrary, a narrow genetic bottleneck is found in modern cultivars [11]. Landraces are the heritage of generations of farmers, reflecting not only the plurality of the landscapes but also of old farmer’s production systems. Landraces were shaped both by edaphoclimatic and traditional agrarian systems diversity and by plurality of human customs. In the last decade, there has been a rapid evolution in cherry cultivation, which has fostered new interest for this highly appreciated crop. New high-quality varieties with improved taste, fruit size, productivity, and, to a lesser extent, resistance to biotic and abiotic stresses, have been developed. For a long time, a small number of sweet cherry varieties (such as ‘Burlat’, ‘Bing’ or ‘Summit’) dominated the market. However, a much wider range of varieties, spanning the whole range of maturity period, have been recently released. Nevertheless, molecular diversity studies conducted with simple sequence repeats (SSR) have demonstrated the narrow genetic base that has been used up to date for the breeding of modern cherry varieties [11–13]. Moreover, the main production regions base their production on a very restricted number of varieties (i.e.: in Turkey, the main world producer, 90 % of the sweet cherry production is assured by ‘0900Ziraat’ cultivar [14]).

In Europe, cherry producers face nowadays new challenges such as sustainable production of high quality fruits, climate change or invasion of new pathogens (i.e. *Drosophila suzukii*). Hence, exploring cherry genetic diversity is crucial in order to create new cultivars well adapted to these challenges. *Ex situ* genetic resources collections remain valuable reservoirs of allelic variability for many traits not yet exploited in current breeding programs. Cherry collections characterization is therefore a major step to facilitate the increased utilization of cherry genetic resources and encourage the sharing of conservation responsibilities between countries in Europe. INRA is the leader of the *Prunus* genetic resources French national network and it manages large cherry collections including the French National Sweet Cherry collection. The preservation, evaluation and management of large *ex situ* germplasm collections are expensive and time consuming [15, 16]. Hence, identifying ‘core collections’ that maximize cherry genetic diversity with minimum redundancy represents a suitable solution to reduce costs. In addition, ‘core-collections’ may be useful tools as a first step in genetic association studies [17, 18]. Criteria based on genetic distances between accessions have been shown to be ideal for evaluation and creation of ‘core collections’ [19]. Knowledge of the genetic structure of heterogeneous germplasm collections is essential when forming core collections [16] and is a prerequisite for deciphering complex traits in genetic resources using association mapping [20]. Association mapping is based on the nonrandom association of alleles at two or more loci, named linkage disequilibrium (LD). Linkage disequilibrium has been estimated in sweet cherry, using relatively few SSRs, showing a medium decay compared with self-compatible peach [21]. To our knowledge, no previous study examined the extent of LD in sweet cherry germplasm with a high number of genome-wide distributed markers. In addition, medium-density SNP arrays have not previously been evaluated for characterizing genetic diversity, population structure and construction of core collections in sweet cherry.

In the context of association mapping, the identification of subgroups within a population or within germplasm collections is a condition for the unbiased estimation of association parameters [22]. In most instances, population’s heterogeneous structure reflects adaptation, domestication, and/or breeding effects. In *Prunus avium*, previous studies have shown a marked genetic bottleneck between wild and cultivated cherries [11, 23] as well as a population structure showing three clusters: wild cherry, landraces, and modern sweet cherry cultivars [11].

Here, we investigated 210 accessions of the INRA’s cherry genetic resources collection with the medium-density RosBREED 6 K SNP array [24]. The objectives of this study were: i) to evaluate the genetic diversity and to estimate the levels of population structure ii) to detect the patterns of LD on cherry and iii) to identify a ‘core collection’ suitable for association genetic studies.

## Methods

### Plant material

The sweet cherry collection studied is maintained by the INRA’s *Prunus* Genetic Resources Center at Bourran (Lot & Garonne), near Bordeaux (France). A total of 210 accessions were studied, 50 % of them are of French origin, and belong for a large part to the French National Sweet Cherry Genetic Resources Collection. The rest of the accessions are of 15 other countries of America, Asia and Europe, with a total number of accessions per country ranging from one to twenty (Additional file 1: Table S1). The accessions can be divided into landraces (*n* = 99) and bred cultivars. Bred cultivars (*n* = 111) result from selections made quite early (*n* = 27) and from modern breeding (*n* = 84). This classification was mainly based either on information coming from literature or, for the French National Sweet Cherry collection, on information gathered in collaboration with the ‘Centre National de Pomologie’ at Alès (Gard, France) (http://pomologie.ville-ales.fr/). Six Spanish landraces and one Hungarian modern variety, not included in the INRA’s *Prunus* Genetic Resources Center, were included in the study and were provided by PhD Angel Fernandez i Marti (Additional file 1: Table S1). One accession by cultivar was studied excepted for two cultivars ‘Noir d’Ecully’, and ‘Giorgia’ for which two accessions of each were studied, corresponding to different introduction periods.

### DNA extraction

Leaf material was frozen in liquid nitrogen and stored at −80 °C for later use. Genomic DNA was extracted from the frozen tissue using the DNeasy® plant kit (Qiagen, Hilden, Germany) according to the manufacturer’s instructions. Genomic DNA was quantified using spectrophotometry Nanoview (GE Healthcare) and fluorimetry Quant-iT™ Picogreen® (Invitrogen) according to the manufacturer’s instructions. Fifteen μl of DNA with a concentration between 50 ng/μl – 75 ng/μl were used for subsequent analyses.

### SNPs genotyping

All accessions were genotyped using the RosBREED cherry 6 K Illumina Infinium II® SNP array v1 [24]. Genotype differences were recorded in the iSCAN platform and SNP genotypes were determined using Genome Studio Genotyping Module (Version 1.8.4, Illumina™) as described in [24]. The RosBREED cherry 6 K SNP array v1 markers used in this work were deposited in NCBI’s dbSNP repository available at www.ncbi.nlm.nih.gov/projects/SNP [25] and each SNP was given a unique accession number that starts with the prefix ‘ss’ (SNPs NCBI ss# database names). More information associated with these SNPs is available at the Genome Database for Rosaceae (GDR; www.rosaceae.org [26]). Physical positions of the SNPs [24]were inferred from the peach genome [27] and the macrosynteny of peach-sweet cherry genomes [28]. SNP positions of the ROSBREED cherry 6 K array v1.0 on the peach genome v2.0 were redefined using batch BLAST function available at the GDR’s website (GDR; www.rosaceae.org [26]) (Additional file 1: Table S2).

Illumina’s GenCall software algorithms for clustering, calling and scoring genotypes were first used to assure SNP quality. SNPs below 0.2 10 %-Gen-Call were removed. Initial clustering was done using Gentrain2, a GenomeStudio build-in clustering algorithm [29]. Following the clustering by Gentrain2, all SNPs were visually examined for appropriateness of clustering, cluster separation, number of clusters, presence of null alleles and paralogs. A SNP was considered ‘failed’ if it showed (1) overlapping clusters or ambiguous clusters which could not be improved by even manual clustering (2) more than 3 clusters suggesting presence of paralogs or (3) very low call frequency [29]. The failed SNPs were not used for further analysis. SNP markers with missing data above 5 % were also discarded for further analysis.

### Analysis of genetic variation

The Hardy Weinberg equilibrium (HWE) and the minor allele frequency (MAF) were calculated for each SNP using PLINK [30]. The SNPs showing severe distortion of the HWE (*p* < 10e-4), or MAF lower than 0.05, were discarded from further analysis.

The average number of alleles, the observed heterozygosity (H_o_), the expected heterozygosity (H_e_) and the inbreeding coefficient (F_IS_) were calculated on landraces and bred cultivars using adegenet 2.0 R package [31, 32].

### Bottleneck detection

We tested for recent population bottlenecks in the three groups of plant material (landraces and early and modern breeding) using BOTTLENECK v1.2.02 program [33]. A Sign test and a Standardized differences tests under a two-phase mutation (TPM) model [34] was used to determine whether population clusters had undergone a recent bottleneck.

### Linkage disequilibrium

Because LD can affect both Principal Coordinate Analysis (PCoA) and STRUCTURE analysis, the marker set was pruned by excluding SNPs in strong LD using PLINK software [30]. SNPs were pruned with a window of 50 SNPs and a step size of 5 makers. The r^2^ threshold was 0.5. Pairwise LD measures for multiple SNPs were calculated using PLINK [30].

Correlations based on genotype allele counts, i.e. not phased genotypic data, were used to estimate the LD using PLINK [30]. The squared correlation based on genotypic allele counts is therefore not identical to the r^2^ as estimated from haplotype frequencies, although it will typically be very similar. Because it is faster to calculate, it provides a good way to screen for strong LD [30]. Total length of each chromosome was chosen as window size and all SNP pairs were reported within each chromosome. The relationship between LD decay and genetic distance was summarized by fitting a locally-weighted linear regression (loess) line to r^2^ data [35] using R function ‘loess’ [36]. r^2^ summarizes both recombinational and mutational history [37].

### Population structure

PCoA (also referred to as Classical Multidimensional Scaling), Bayesian-based (STRUCTURE software [38]) and Discriminant Analysis of Principal Components (DAPC) analysis were used to investigate the pattern of population structure.

PCoA is a distance-based model which uses jointly a dissimilarity matrix calculated with a simple-matching index, and a factorial analysis. PCoA was performed using DARwin 6.0.010 software (Dissimilarity Analysis and Representation for Windows) [39, 40]. This software produces graphical representations on Euclidean plans which preserve at best the distances between units [39, 40].

The model-based approach implemented in the software package STRUCTURE [38] was also applied to infer population structure. Structure software options offers to split the Graphic User Interface from the main algorithm helping to set large numbers of runs on a computing cluster (Additional file 2: Figure S1). According to this useful scalability, this study supported more than 10,000 CPU hours, tests and benchmarking operations included. Computer time for this study was provided by the computing facilities MCIA (Mésocentre de Calcul Intensif Aquitain) of the Universities of Bordeaux and Pau et des Pays de l'Adour. Twenty runs of STRUCTURE were done by setting the number of clusters (K) from 1 to 16 (number of countries of origin of the sampled accessions). Each run consisted of a burn-in period of 10.000 steps followed by 100.000 Monte Carlo Markov Chain (MCMC) replicates, assuming an admixture model and uncorrelated allele frequencies. No prior information was used to define the clusters. For the choice of the most likely number of clusters (K), the plateau criterion proposed by Pritchard et al. [38] and the ∆K method, described by Evanno et al. [37] and implemented in Structure Harvester [41], were used. In order to assess assignment success, STRUCTURE was run by enforcing K to its true value. For a given K, we used the run that had the highest likelihood estimate to assign cluster proportions to individuals. Accessions with estimated memberships above 0.8 were assigned to corresponding groups whereas accessions with estimated memberships below 0.8 were assigned to a mixed group. We ran STRUCTURE on partitioned datasets in order to investigate lower levels of structure, in relation to the results obtained. For the partitioned datasets, K was allowed to vary from one to four for the ‘Bred cultivars’ subgroup and from one to 11 for the ‘Landraces’ subgroup, in agreement with the number of countries of origin of the accessions in each subgroup. Pairwise F_st_ [42] among the subpopulations identified by STRUCTURE were calculated using adegenet 2.0.

The assumptions underlying the population genetics model in STRUCTURE may limit its use in crops. Unlike natural populations, crops are subjected to displacements, breeding, clonal propagation, absence of panmictic conditions. Thus, we complemented the STRUCTURE analysis with the DAPC. The absence of any assumption about the underlying population genetics model, in particular concerning Hardy-Weinberg equilibrium or linkage equilibrium, is one of the main assets of DAPC analysis [43]. DAPC was used to identify and describe clusters of genetically related individuals, as implemented in the R’s package adegenet 2.0 [31, 32]. DAPC transforms the data using PCA, and then performs a Discriminant Analysis on the principal components (PC) retained using a cross-validation method. This multivariate method is suitable for analyzing large numbers of genome-wide SNPs, and it provides individuals’ assignment to groups as well as a visual assessment of between-population differentiation.

The number of PCs retained can have a substantial impact on the results of the analysis. Indeed, retaining too many components with respect to the number of individuals can lead to over-fitting and instability [31]. We used the optimization procedure proposed by the R’s package adegenet to assess the optimal number of PCs to be retained [32]. The cross-validation procedure implemented with the function xvalDapc performs stratified cross-validation of DAPC using varying numbers of PCs (and keeping the number of discriminant functions fixed) [31]. Pairwise F_st_ [42] among the DAPC clusters were calculated using adegenet 2.0.

### Core collection creation

Core collections are subsamples of larger genetic resources collections which are created in order to include a minimum number of accessions representing the maximum diversity of the original collection. DARwin 6.0.010’s function ‘maximum length sub tree’ has been used to select a reference set in chickpea [44], cowpea [45] and sorghum [46]. DARwin version 6.0.010 was used to build the diversity trees [39, 40]. Dissimilarities were calculated with 10.000 bootstraps and transformed into Euclidean distances. Un-Weighted Neighbor-Joining (N-J) method was applied to the Euclidean distances to build a tree with all genotypes. Then, ‘maximum length sub tree function’ was used to draw the core collection. Maximum length sub-tree implemented is a stepwise procedure that successively prunes redundant individuals. This procedure allows the choice of the sample size which retains the largest diversity, and is visualized by the tree as built on the initial set of accessions (210 accessions in this case). Two accessions are redundant if their distance in the tree, as judged by the edges length, is small. The accessions with the longest edge have more uncommon characters and are therefore genetically most diverse. Putative clusters of synonym accessions were identified using ‘removed edge value’ provided by the NJ tree. A threshold value of 0.0008 was chosen to identify putative synonyms. Sphericity index and the length of pruned edge of the initial tree length were used to choose the final core collection accounting for maximum genetic diversity [39, 40].

### Availability of supporting data

The genotyping data set supporting the results of this article are available at https://www.rosaceae.org/ and at INRA’s GnpIS repositories {Steinbach, 2013 #3425}.

## Results

### SNP genotyping and variation

The genotyping of 210 landraces and cultivars with the RosBREED Cherry 6 K SNP array generated genotyping data points (Table 1). After removal of SNPs failing to generate clear genotype clustering (Illumina™ GenCall 10 % lower than 0.2), 5186 SNPs with high quality genotype calls were obtained. SNP markers with missing genotypes above 5 % were deleted. Markers showing high distortion for Hardy-Weinberg equilibrium (>0.0001) (*n* = 40 SNPs) or Minor Allele Frequency (MAF) (*n* = 3269 SNPs) lower than 5 % were discarded for further analysis using PLINK [30]. Homozygous markers for all the individuals (*n* = 2785 SNP) were deleted in the MAF step. A total of 1215 SNP markers were retained after these filtering steps (Table 1). These 1215 SNPs markers were distributed over the eight chromosomes with a median distance between markers of 96 kb and an average of 152 SNP markers per chromosome. The largest gap (3.6 Mb) was located in LG3 (Additional File 2: Figure S2). SNP markers were LD pruned before performing PCoA and STRUCTURE analysis to avoid bias using PLINK [30]. 889 SNP markers were deleted and a total of 326 SNPs were retained (Table 1). These 326 SNPs markers were distributed over the eight chromosomes with a median distance between markers of 463 kb and an average of 41 SNP markers per chromosome. The largest gap (7.8 Mb) was located in LG2 (Additional File 2: Figure S2).

**Table 1:** Quality filtering of SNPs

Criteria	Threshold	Total SNP	Deleted SNP	Conserved SNP
GenCall 10 %	<0.2	5696	510	5186
Missing data	>5 %	5186	662	4524
HWE	>0.0001	4524	40	4484
MAF^a^	<0.05	4484	3269	1215
LD (VIF)	2	1215	889	326

^a^Includes homozygous SNP

GenCall 10 % from Illumina^TM^, missing data, Hardy Weinberg equilibrium (HWE), minor allele frequency (MAF) and linkage disequilibrium (LD) (VIF -variance inflation factor -)

### Estimation of genetic diversity

The average number of alleles in both early and modern cultivars combined (bred cultivars) was the same than in landraces, whereas the number of alleles was lower in early selections than in modern breeding cultivars (Table 2). This could be associated to the lower number of early selections (*n* = 27), as compared to the modern breeding sample (*n* = 84).

**Table 2:** Genetic diversity estimations in landraces and bred (early and modern) cultivars in sweet cherry

Classification	Statistic	Number of individuals	Number of alleles	Ho	He	Fis
Landraces		99	652	0.316	0.303	−0.0424
Bred cultivars (early and modern)		111	652	0.298	0.275	−0.08312
	*t*-test			^a^	^a^	^a^
	*p*-value			0.001	0.000	2.62E-06
Early selections		26	646	0.313	0.278	−0.12418
Modern breeding		85	651	0.294	0.269	−0.08944
	*t*-test			ns	ns	ns
	*p*-value			0.010	0.078	0.4418

^a^, ns: significant or non-significant differences at 99 % confidence interval, respectively

Genetic diversity parameters showed higher diversity in landraces compared to bred cultivars. However, no significant differences in observed or expected heterozygosity were found between modern and early selected cultivars. Further, inbreeding was lower for landraces compared to bred cultivars (both early and modern), whereas no differences were found between early and modern cultivars (Table 2).

### Bottleneck detection

To verify whether the landraces, early and modern bred cultivars have experienced a population reduction in size, we detected excess heterozygosity in a population at mutation-drift equilibrium (H_eq_) under the two-phase mutation (TPM) model [47] by using the program BOTTLENECK. Landraces, early and modern bred cultivars showed significant (*P* < 0.01) heterozygosity excess under the model as an indication of recent demographic contraction.

### Linkage disequilibrium

**Fig. 1:**
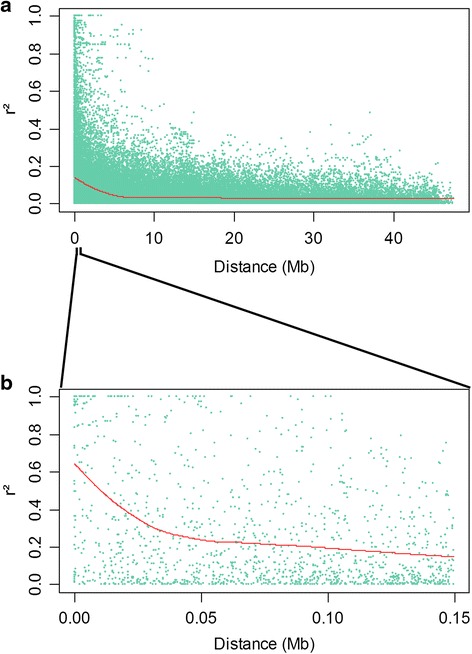
Linkage disequilibrium decay. Scatter plot of LD decay (r^2^) against the genetic distance for pairs of linked SNP across the eight linkage groups (**a**). Zoom-in scatter plot of LD decay (r^2^) against the genetic distance (**b**). Distance (Mb) is estimated from peach genome v2.0 [27] and high macrosynteny found between peach and sweet cherry [28]

Detailed understanding of the linkage disequilibrium in a population of cultivars is crucial when considering the application of association genetics or GWAS in a species. In this study, the extent of LD was evaluated in 210 *P. avium* trees using 1215 non LD-pruned SNP markers (Fig. 1). The overall LD estimated in our plant material was very low and few values of r^2^ > 0.8 were found (Fig. 1a). On average, intra-chromosomal LD declined below r^2^ = 0.2 at around 0.1 Mb (Fig. 1b).

### Population structure

The genetic structure of the INRA’s Sweet Cherry genetic resources collection was analyzed using STRUCTURE, PCoA and DAPC. All analyses were performed with the LD-pruned 326 SNP set.

**Fig. 2:**
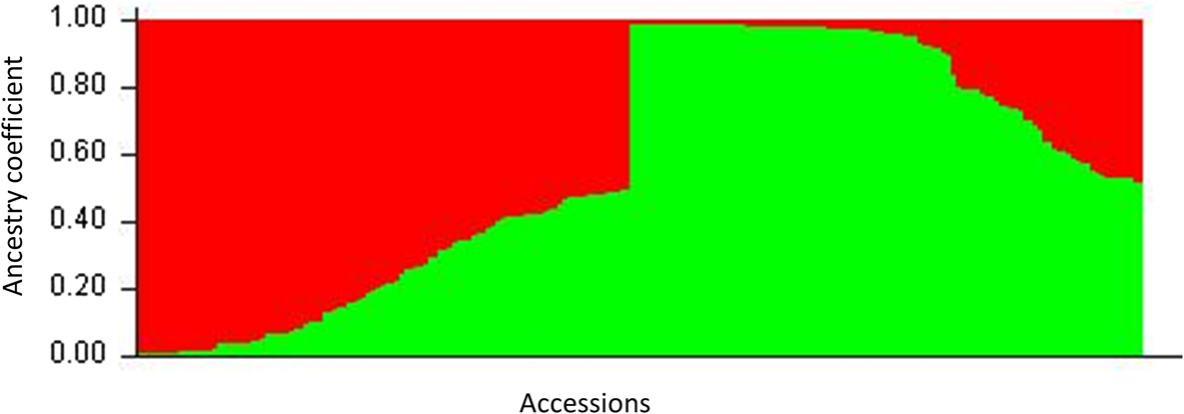
Inferred population structure of the collection using STRUCTURE software. Bar plot of individual ancestry proportions for the genetic clusters inferred using STRUCTURE (K = 2) and the reduced dataset (326 SNP data). Individual ancestry proportions (q values) are sorted within each cluster. Admixture model, independent frequencies, 10,000 burn-in iterations, 100,000 Markov Chain Monte Carlo iterations were used for this analysis. Bred cultivars and landraces ancestral populations are shown in green and red, respectively

**Fig. 3:**
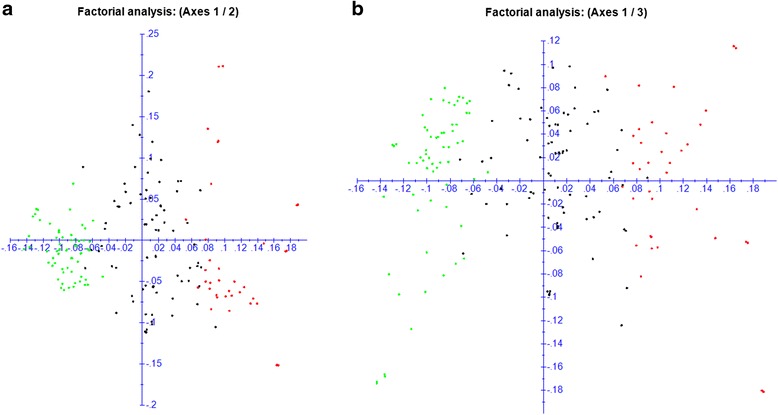
Principal coordinates analysis (PCoA). PCoA using 326 selected SNP with no linkage disequilibrium in the set of 210 sweet cherry accessions. Landraces cluster identified in STRUCTURE is shown in red, bred cultivars cluster in green and admixed cluster in black. First and second components (**a**) and first and third components (**b**) of the PCoA analyses are shown

Thanks to the scalability of STRUCTURE software and MCIA multi-core infrastructure, we reduced the computing time from one year to few days. In STRUCTURE the most likely number of clusters was evaluated considering the ∆K method [48] and the plateau criterion [38]. The ∆K criterion gave the highest value for K = 2 (Additional file 2: Figure S3; Additional file 1: Table S3). This method is known to give rise to the first structural level in the data, here two ancestral populations were identified (Fig. 2). The first one (referred as ‘Landraces’ from now on) accounts for 50 accessions, from which 76 % are landraces, whereas the second population (referred as ‘Bred cultivars’ from now on) comprises 71 accessions, from which 74 % are bred cultivars resulting from both early selection in the 19th century and modern breeding. In addition, a large number of accessions (*n* = 88, e.g. about 50 % of the collection) showed mixed ancestry (membership values lower than 80 % in any of the two clusters). In the admixed cluster, landraces and early selected or modern bred accessions are equally represented. The majority (*n* = 12) of the 18 Italian accessions (all bred cultivars) of the INRA’s collection showed mixed ancestry, among them only ‘Adriana’ has a membership value lower than 50 % in the bred cluster. Nearly 53 % of the French bred cultivars are admixed, 62 % of them being selections from the INRA’s sweet cherry breeding program: ‘Ferbolus’, ‘Fernier’, ‘Fercer’, ‘Ferprime’ and ‘Folfer’, showing more than 50 % of membership in the bred cluster. Results obtained with STRUCTURE were confirmed by the representation of PCoA analysis based on genetic distance matrix using DARwin 6.0.010 software [40] (Fig. 3). Cherry accessions formed two main clusters corresponding to the two ancestral populations identified with STRUCTURE. The landraces cluster was more scattered than the breeding cultivar one. The admixed accessions were dispersed between these two clusters along the axis 2 (Fig. 3). Pairwise F_st_ values among STRUCTURE clusters ranged from 0.022 (Admixed-Bred cultivars) to 0.058 (Landraces-Bred cultivars) (Additional file 1: Table S5).

As the Evanno ∆K preferentially detects the uppermost level of structure of the data [47], we analyzed each cluster independently to explore whether a substructure could be detected within each group. The two partitioned datasets comprised 72 accessions of the ‘Bred cultivars’ ancestral population and 50 accessions of the ‘Landraces’ ancestral population. The 88 accessions considered as admixed were discarded from further analyses. Within the two groups, ‘bred cultivars’ and ‘landraces’, STRUCTURE allowed the identification of two subgroups in each group (Additional file 1: Table S4). ‘Bred cultivar’ group was separated in two clusters. The first one is formed by 63 % of the total bred accessions (cluster: Bred cultivars 1) and it includes most of the American (from the USA and Canada) and French modern varieties hosted in the INRA’s sweet cherry genetic resources collection. The second cluster is smaller, 11 % of the total bred accessions (cluster: Bred cultivars 2), and consists mainly in European accessions, the Iranian cultivar ‘Noire de Meched’ and ‘Stark Lambert’ from USA. The admixed group contains all the Eastern European modern varieties with the exception of ‘Badacsony’ accession, which was included in the ‘Bred cultivar 2’ group.

Concerning the landraces group, the Evanno criterion gives a strong signal for K = 2 and a weaker for K = 4 (Additional file 1: Table S4). When K = 2 was considered, landraces were split into two clusters. The first one contained 34 % of the total number of landraces accessions (cluster: Landrace 1) and it gathered accessions from Spain, Hungary, Great Britain and France, including ‘Early Burlat’. The second one included 12 % of the total number of landraces accessions (cluster: Landrace 2), which were all of French origin. Remaining landraces accessions (54 %) were admixed.

**Fig. 4:**
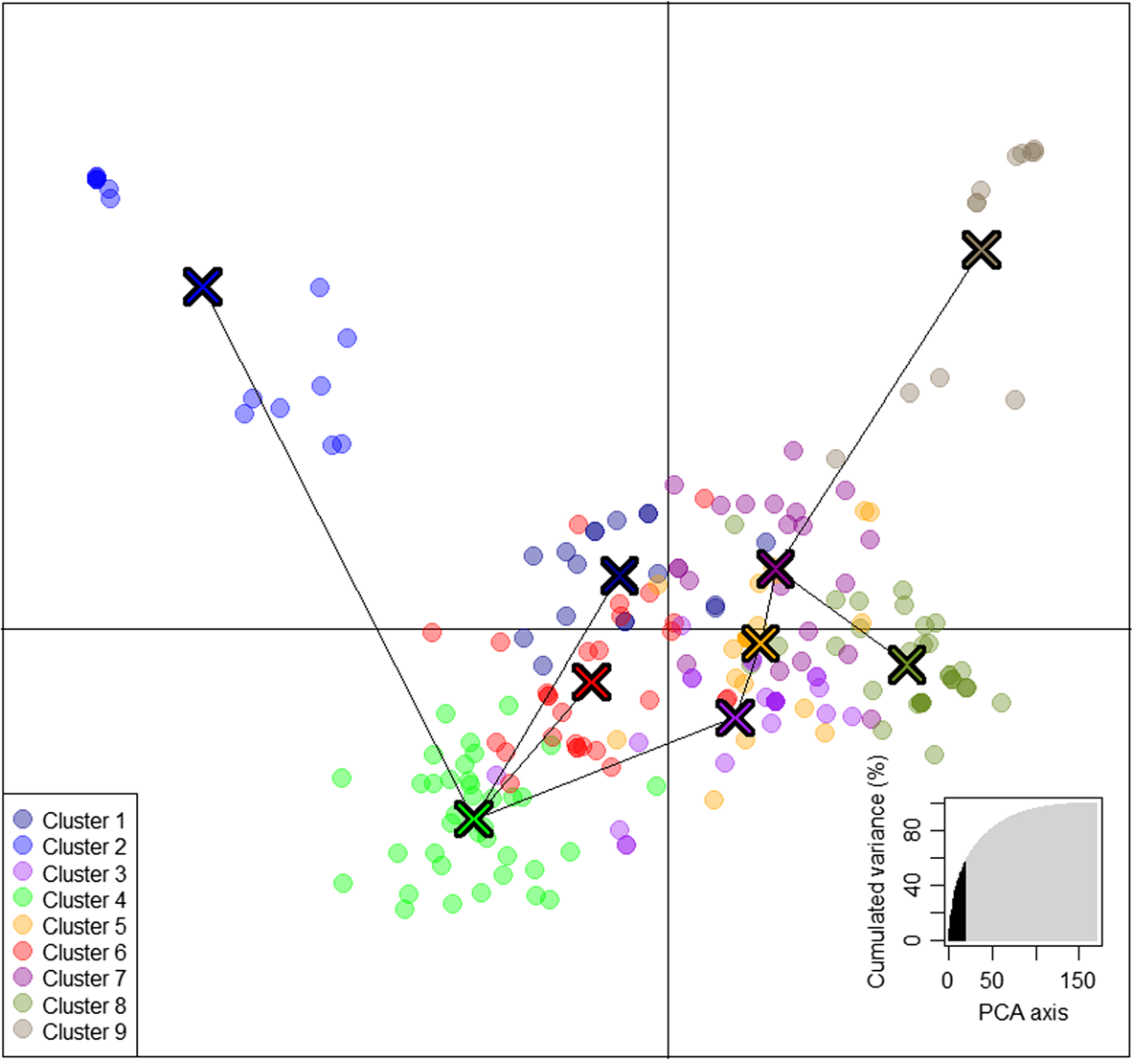
Discriminant analysis of principal component (DAPC) scatter plot of individuals using the 326 SNP set. 20 PCs (Additional file 2: Figure S5) and eight discriminant functions (dimensions) (Additional file 2: Figure S8) were retained during analyses, to describe the relationship between the clusters. The scatterplot shows only the first two PCs of the DAPC analysis. The bottom right graph illustrates the variation explained by the 20 PCs

The second criterion used to evaluate the most likely number of clusters was the plateau criterion [38]. Here, the mean log-likelihood curve attained a maximum value around K = 9; beyond this value, it decreased slightly before reaching a plateau, showing an increase of the associated estimates’ standard deviation (Additional file 2: Figure S4). To cross-check the results from STRUCTURE with a model-free method, a third method, DAPC, was used. The functions ‘find.clusters’ and ‘*k*-means’ algorithm were used to determine the number of clusters maximizing the variation between clusters [31]. To avoid the loss of information these two functions were performed with 170 Principal Components, accounting for more than 98 % of the variance (Additional file 2: Figure S5). The Bayesian Information Criterion (BIC) was used to identify the optimal number of clusters, 9, indicated by an elbow curve of BIC values as a function of *k* (Additional file 2: Figure S6). The number of retained PC for DAPC analyses was calculated using a cross validation method implemented in ‘xvalDapc’ function from R adegenet 2.0 package [31, 32]. ‘xvalDapc’ function minimized the mean square error using 20 PC (Additional file 2: Figure S7). Also, a bar plot of eigenvalues for the discriminant analysis was used to select eight discriminant functions to be retained (Additional file 2: Figure S8). Thus, a scatter plot was drawn using nine clusters obtained by BIC, 20 PCA obtained by xvalDapc, and the two main axes of the discriminant analysis (DA) (Fig. 4). Pairwise F_st_ values among DAPC clusters ranged from 0.043 (Cluster 4-Cluster 6) to 0.142 (Cluster 2-Cluster 9) (Additional file 1: Table S6). Membership values of each individual to the nine clusters are available in the assign-plot (Additional file 2: Figure S9). Clusters 2, 4 and 9 were clearly differentiated using the two main DA eigenvalues (Fig. 4). Cluster 2 consisted in accessions mainly released by breeding programs from Eastern European countries (e.g. Hungary and Romania). It also includes the German variety ‘Regina’ and the set of accessions: ‘Badacsony’, ‘Gégé’, ‘Belge’, ‘Noire de Meched’ and ‘Ferrovia’ (Additional file 1: Table S4). Cluster 4 included only modern varieties. It contains 85 % of Canadian accessions of the INRA’s collection, among which ‘Van’ and some of its descendants (e.g. ‘Lapins’, ‘Summit’, ‘Newstar’, ‘Sumtare’, etc.), 47 % of the American ones in particular ‘Hardy Giant’ and ‘Garnet’, and 61 % of the French ones, with ‘Fercer’ and all its derived hybrids (‘Ferprime’, ‘Ferdiva’, ‘Ferdouce’, ‘Feria’), except ‘Folfer’ and ‘Ferlizac’ which are included in DAPC clusters 3 and 5, respectively. Most of the accessions comprised in cluster 9 are landraces with a short flowering-maturity period.

Clustering performed by DAPC is consistent with the available information on pedigree data (Additional file 1: Table S4). For example, ‘Burlat’ and its descendants clustered together in group 3. Also, DAPC clustering was represented according to the countries of origin (Additional file 2: Figure S10). The plant material analyzed in this study from countries such as Canada, Italy, Spain or USA, showed a narrow genetic diversity, with most of each country’s cultivars included in only one or two clusters. Also, the results confirmed the large diversity of the French germplasm included in all the clusters.

We compared the 9 subgroups obtained from STRUCTURE and DAPC: both approaches provided similar results (Additional file 2: Figure S11a). When admixed individuals were all considered as an admixed group (group 10) in the DAPC analysis, the clusters calculated by STRUCTURE and DAPC analysis were the same, except for STRUCTURE groups one and six, which were included in DAPC group 8 (Additional file 2: Figure S11b).

One interesting feature of DAPC method is that it allows calculating the contributions of alleles to the regions of the genome driving genetic divergence among groups [43]. However, no significant allele contribution (named as loading) was found for the main two dimensions on our analysis (Additional file 2: Figure S12).

DAPC was also performed by using 1215 SNPs as no assumption on LD equilibrium is required for DAPC analysis [43]. The same number of clusters (nine) was obtained. Most of the individuals clustered in the same clusters as in the 326-SNP DAPC analysis. However, individuals showing a low membership value (homologous to the admixture coefficients from STRUCTURE) were clustered to different groups compared with the 326-SNP DAPC analysis (Additional file 2: Figure S13). Clustering performed slightly better with the 326 than with the 1215 SNP set, obtaining higher membership scores for the defined clusters.

### Core collection

The aim of developing genetic core collections is to select a reduced set of accessions representing the genetic diversity among individuals in a large source of germplasm. A first core reference set, suitable for association genetic studies, was selected to capture the genetic diversity of sweet cherry available in the INRA’s Sweet Cherry Collection.

**Fig. 5:**
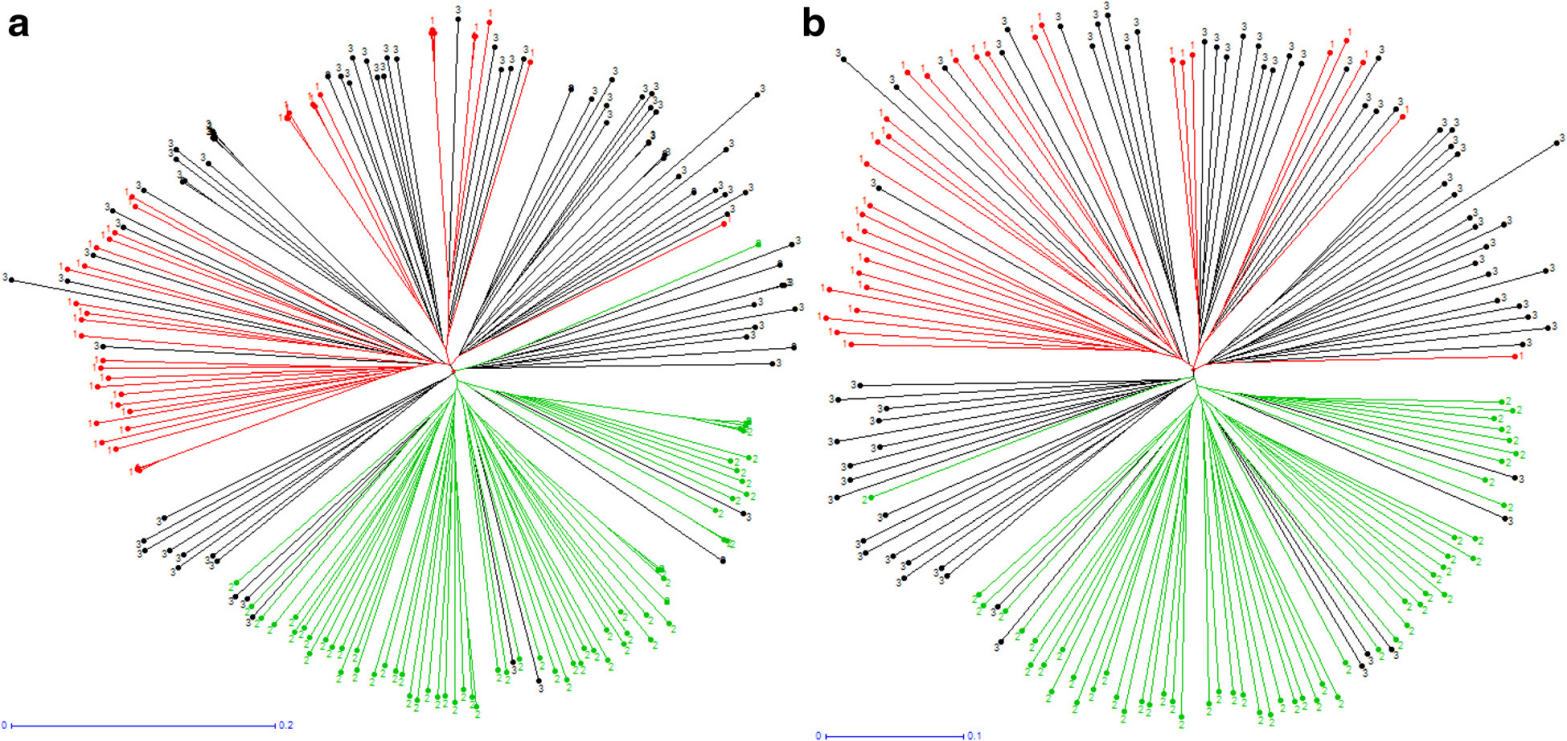
Neighbor-Joining Trees compared with STRUCTURE results (K = 2). Trees from SNP data of the INRA’s sweet cherry collection (**a**) and the constructed core collection (**b**). Colors indicate the clusters calculated using STRUCTURE: landraces (red, number 1), bred cultivars (green, number 2) and admixed (black, number 3)

**Fig. 6:**
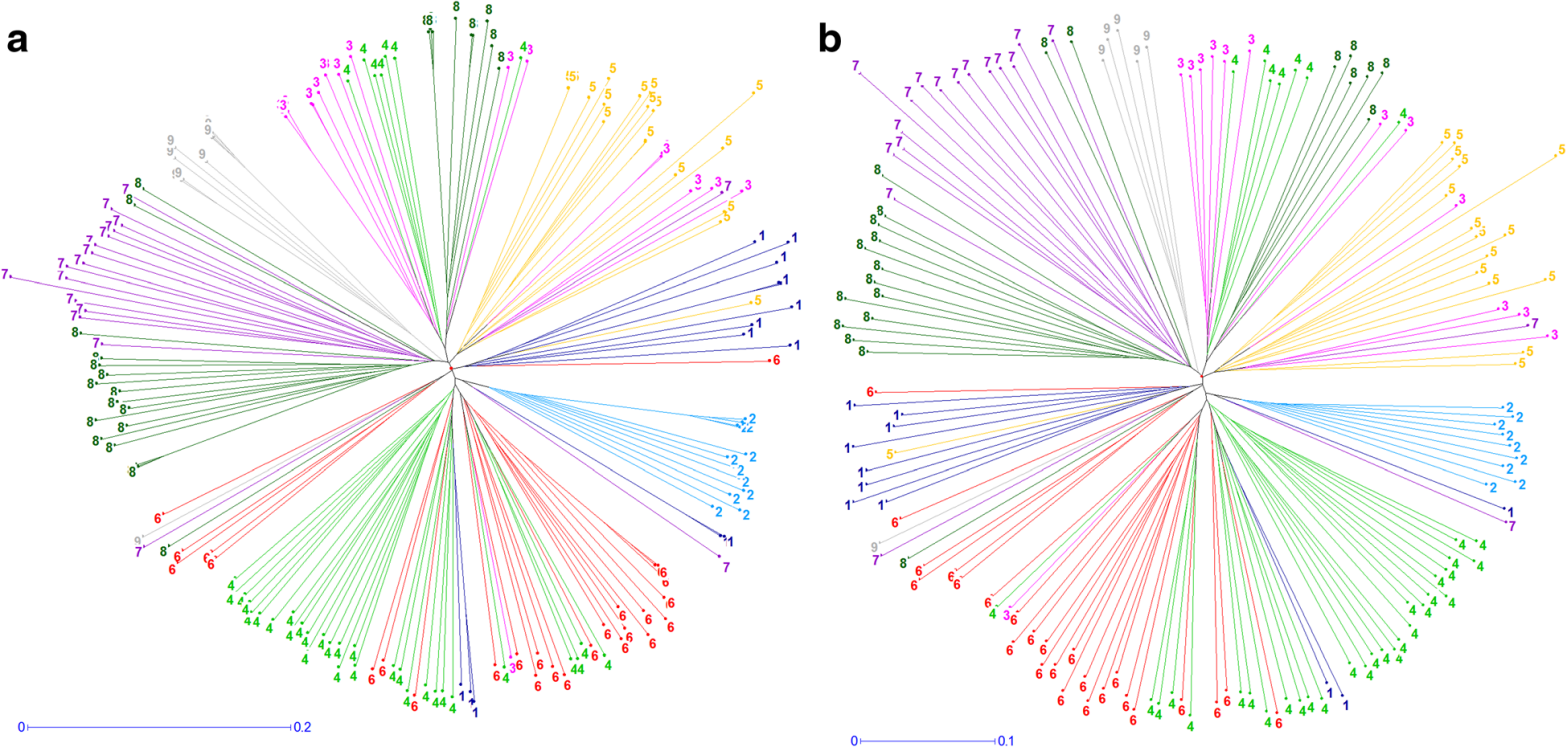
Neighbor-Joining Trees compared with DAPC results. Trees from SNP data of the INRA’s sweet cherry collection (**a**) and the constructed core collection (**b**). Colors indicate the clusters (K = 9) calculated using DAPC: Cluster 1 is dark blue, Cluster 2 is light blue, Cluster 3 is pink, Cluster 4 is light green, Cluster 5 is orange, Cluster 6 is red, Cluster 7 is purple, Cluster 8 is dark green and Cluster 9 is grey. Detailed information of the composition of each cluster is provided in Additional file 1: Table S4

Neighbor-joining (NJ) tree based on the dissimilarity matrix between 210 accessions of the INRA’s Sweet Cherry Collection was initially built to assess the genetic distribution of markers. Groups of NJ tree were, in general, in agreement with STRUCTURE (K = 2) (Fig. 5a) and DAPC analysis (K = 9) (Fig. 6a), although some individuals were assigned to different clusters depending on the approach.

DARwin 6.0.010 function maximum length sub tree method was iteratively used to eliminate the most redundant accessions until the percentage of sphericity index and pruned edge came to a flat line, corresponding to 156 accessions (Additional file 1: Table S4; Additional file 2: Figure S14).

Putative clusters of synonym accessions were identified using removed edge value of NJ tree. A total of 48 accessions were grouped in 17 groups of synonymy (Additional file 1: Table S4). Putative synonym groups included from two to six individuals. For example, ‘Michaude’, ‘Bigarreau Hâtif Burlat’, ‘Beaulieu’, ‘Lazar’, ‘Bigarreau Semi-Hâtif’ and ‘Ogier’ were identified as putative synonyms. Moreover, two accessions corresponding to different introduction periods of both ‘Noir d’Ecully’ and ‘Giorgia’ cultivars, were proved to be identical using the RosBREED cherry 6 K SNP array v1.

## Discussion

### SNP genotyping and variation

This study provides the first overview of the genetic variation in a large collection of sweet cherry germplasm using a medium-density array of SNP genome-wide distributed markers. We provide the first study confirming the utility of the RosBREED array v1.0 [24] for genotyping a collection of genetic resources of *P. avium*. Despite the use of a relatively low number of detection panel accessions, each sequenced at low depth, for the development of the RosBREED array v1.0 [24], we obtained more than 1200 high-quality SNPs after filtering. This array of SNP markers allowed to increase the marker density compared with previous diversity analyses performed with SSR or SNP markers in sweet cherry [11, 21, 49].

### Estimation of genetic diversity

Observed and expected heterozygosities calculated in this study were lower than those calculated using SSRs in cultivated sweet cherry [11, 50]. This can be related to the lower information provided by SNPs compared to SSRs for variability studies, as shown in peach [51]. Also, Ho and He were slightly lower in our data than in a panel of 36 sweet cherry accessions using 76 SNP markers (Rosaceae Conserved Orthologous Set) [52]. The excess of heterozygosity (Table 2) can be linked to the Gametophytic Self-Incompatibility (GSI) system controlling sexual reproduction in sweet cherry [53].

Significant differences for H_o_, H_e_ and inbreeding coefficient (F_IS_) between landraces and selected cultivars are in agreement with the loss of diversity associated to breeding. A higher impact of breeding compared to domestication was shown for sweet cherry [11]. However, in our study no significant differences between modern and early selections were observed. This could be due to the low number of individuals available in these groups, especially in the ‘Early selections’ group.

### Bottleneck detection

The excess of heterozygosity found for sweet cherry can also be related to a genetic bottleneck, as found by the BOTTLENECK software. These results are in agreement with previous bottleneck events suggested in sweet cherry [4, 11]. When a population experiences a reduction of its effective size, it generally develops a heterozygosity excess at selectively neutral loci [47].

### Linkage disequilibrium

When LD declines rapidly with distance, LD mapping is potentially very precise [54]. LD decays more rapidly in cross-pollinated species as compared to self-pollinated species because recombination is less effective in the latter. LD can also be related to reduction in population size accompanied by extreme genetic drift [55]. Selection produces bottlenecks at a specific locus and those linked. In addition, selection for epistatic loci might result in LD of loci not physically linked [37].

LD decays rapidly in a gene by recombination after selection for a particular allele [56], the time scale of domestication (~9000 years ago in maize [57]) may be such that an appreciable selective effect on LD remains [54]. This remained LD could be more important in sweet cherry considering the lower number of recombination events, due to its long cycle and its vegetative propagation through grafting.

The high proportion of SNP loci pairs in LD as well as the decay of LD with distance shows that association mapping is a potential tool applicable to sweet cherry breeding. These results are in agreement with the rapid LD decay previously showed in cultivated sweet cherry using 35 SSR [21]. However, a low proportion of linked SNP pairs with *r*^2^ values *>* 0.8 was found (Fig. 1). Such high r^2^ values are required to detect SNP-phenotype associations explaining low values of phenotypic variance [58]. Thus, a genome-wide association mapping aiming at explaining low percentages of phenotypic variance would need a higher number of markers compared to the SNPs available in the RosBREED cherry 6 K SNP array v1.

Our results show a lower linkage disequilibrium compared to the model species in *Prunus*, *Prunus persica* L., [59]. This can be related to the self-incompatibility system described in sweet cherry [53].

### Population structure

The different approaches (STRUCTURE, PCoA and DAPC) used to analyze the structure of the INRA’s Sweet Cherry collection appeared to provide complementary information. STRUCTURE performed well in detecting global clusters of diversity and results were confirmed by PCoA. Nevertheless, the two parameters used to choose the most likely number of clusters in STRUCTURE did not give the same value for K. Evanno ΔK method gave K = 2 in the whole analysis as well as in the investigation of cryptic structure. The Evanno method finds the uppermost level of structure in the data, as it focuses exclusively on the change in slope. According to some authors this may cause ΔK to be artificially maximal at K = 2 in some cases [60]. Nevertheless similar results were obtained on a previous sweet cherry structure study based on SSR [11], and K = 2 is often reported when analyzing germplasm collections [60–62]. Our results are in agreement with a previous structuration of sweet cherry cultivars into landraces and modern varieties [11]. We completed the analysis using the maximum likelihood parameter as recommended by Pritchard et al. [30], in that case K was set to nine. This value of K appeared to fit with the origin and the pedigree of the accessions.

The DAPC method provides an interesting alternative to STRUCTURE software as it does not require that populations are in HW equilibrium and can handle large sets of data without using parallel processing software. However, as for other multivariate analyses, the reduction of genetic information to interindividual or interpopulation distances may represent a substantial loss of information [63]. Nevertheless, our results showed a good consistency between STRUCTURE and DAPC analyses when no admixed individuals were considered. Also DAPC analysis provided a more detailed clustering within landraces and bred cultivars compared STRUCTURE analysis either in our study or in previous analysis using SSR [11].

Regarding membership to clusters, DAPC provides membership values that are different from admixture coefficients from STRUCTURE, but they can still be interpreted as proximities of individuals to the different clusters [32]. However, group membership provided by R’s adegenet package is more useful for groups defined by external criteria (i.e. biologically) rather than by *k*-means, as *k*-means provides optimal groups for DAPC and therefore both classifications will be mostly consistent [31].

Clustering of individuals presented in this study may give interesting cues for increasing diversity in breeding programs and germplasm collections. For example, landraces were included in all clusters except for cluster four whereas most of modern cultivars were included in only three clusters (four, five and six). This is especially clear for the INRA’s cultivars released in the last two decades, as most of them (more than 60 %) are included in cluster four. Hence, the use of landraces different from the clusters four, five or six, as founding clones, would increase the genetic diversity of new cultivars. Also, most North American cultivars (USA and Canada) are included in two close clusters (four and six). This is in agreement with the repeated use of five founding clones and one genetic source for self-compatibility in sweet cherry breeding in North America [64] and with the lowest F_st_ value found in our study among clusters four and six (Additional file 1: Table S6). This repeated use of a few founding clones and their progeny as parents in breeding programs may eventually result in loss of genetic variability and a concomitant increase in inbreeding depression in future generations [65]. The inbreeding problem and potential genetic limitations have been raised for numerous fruit species modern breeding programs, including sweet cherry [64–66]. A deep knowledge of the structure of the germplasm and the identification of clusters could assist the choice of genitors in current breeding programs, which may maximize genetic diversity and enhance the potential gain from selection. This would help to increase the breeding programs’ efficiency to face new demands from consumers (organoleptic traits) and industry (antioxidant content), as well as new ecological issues (i.e. adaptation to climate change, pest resistances).

### Core collection

Characterization and maintenance of germplasm collections is a laborious task. Genetic and phenotypic knowledge is crucial for a better understanding and utilization of the available genetic resources by breeders [46]. In this study, we propose the first core collection from the INRA’s Sweet Cherry collection, accounting for landraces and cultivars from 16 different countries.

Some putative synonymous cultivars were probably renamed when released in the same region but at different periods of time. For example, ‘Bigarreau Jaboulay’ and ‘Guigne Ramon Oliva’ were released in Southeastern France in 1822 in 1900, respectively. Other possibilities could be that those cultivars were released in different regions or countries, or even commercialized with different names. Thus, ‘Lazar’, described as “a seedling of unknown parentage probably a selection from ‘Burlat’” (Jacques Claverie personal communication) was identified in this study as a putative synonym of ‘Burlat’.

In other context, the cluster of putative synonyms identified in this study: ‘Badacsony’, ‘Belge’ ‘Ferrovia’, ‘Gégé’, ‘Noire de Meched’, and ‘Stark Lambert’; is in accordance with previous fingerprinting analysis using AFLP and SSR markers [67]. However, this clustering is contradictory to the country of origin and the period of release of these cultivars. A comparative study using accessions of these cultivars conserved both in the region of origin (i.e.: Balaton Lac region in Hungary for ‘Badacsony’) and in different institutes is suggested. This recommended study would be essential to elucidate this possible incoherence. In addition, putative synonyms should be verified with a higher density SNP assay or NGS technologies to avoid misassignment. For example, punctual mutation may have not been picked up by the RosBREED sweet cherry array but severely affect the phenotype of an individual. This is the case of two putative synonyms identified; ‘Fougerouse’ and ‘Fougerouse Blanc’ accessions; which show red and yellow fruit color, respectively. ‘Fougerouse’ and ‘Fougerouse Blanc’ represent an excellent material for functional genomics studies aimed at deciphering the fruit color in sweet cherry.

The diversity of INRA’s Sweet Cherry core collection could be maximized by introducing exotic plant material underrepresented so far, such as landraces and wild cherries. For example, the Spanish landraces ‘Punxeta’ and ‘Tarrega’ are two good candidates to be included in the INRA’s Sweet Cherry Collection. In addition

INRA’s Sweet Cherry core collection represent a valuable tool for the development of genome-scale analysis aimed at deciphering the genetic determinism of traits for this species.

## Conclusions

In the present study, we show the first population-genetics analysis in cultivated sweet cherry using a medium-density SNP marker array. We provide estimations of linkage disequilibrium, genetic structure using different approaches and the definition of a first INRA’s Sweet Cherry core collection. This information will be useful for parent selection in breeding programs, germplasm management and association genetics studies. Thanks to the perennial nature of sweet cherry and the ease of vegetative propagation, this core collection could be easily disseminated worldwide for further analyses.

## Additional files

Additional file 1:
**Table S1.** List of accessions including the origin, level of breeding and pedigree. **Table S2.** RosBREED cherry 6 K SNP array 1.0 position using peach genome v2.0 assembly. **Table S3.** Table summarizing the results using Evanno et al. (2005) method (output of Structure Harvester). **Table S4.** List of accessions including membership values to subgroups using STRUCTURE and DAPC analysis. **Table S5.** Pairwise F_st_ calculated among populations identified by STRUCTURE using adegenet 2.0. **Table S6.** Pairwise F_st_ calculated among populations identified by DAPC using adegenet 2.0. (XLSX 341 kb) Additional file 2:
**Figure S1.** Workflow of STRUCTURE 2.3.4 software implementation at MCIA cluster nodes. **Figure S2.** Genome coverage of 1,215 and 326 SNP sets across the eight linkage groups of sweet cherry. **Figure S3.** Graphical method (as in Evanno et al. 2005) allowing the detection of the number of groups K using ΔK. **Figure S4.** Graphical method (as in Evanno et al., 2005) allowing the detection of the number of groups K using the rate of change of the likelihood distribution (Mean log-likelihood values). **Figure S5.** Cumulative variance explained by the principal component analysis (PCA) relative to the number of principal components (PCs) retained in the analysis. **Figure S6.** Selection of the optimal number of clusters in the DAPC using the lowest Bayesian Information Criterion (BIC). **Figure S7.** Cross-validation procedure to choose the optimal number of Principal Components for the DAPC analysis. **Figure S8.** Eigenvalues of retained discriminant functions in the DAPC analysis. **Figure S9.** Assignment plots from DAPC for a K of nine populations. **Figure S10.** Comparison of clustering performed by DAPC (K=9) and origin of cultivars and landraces. **Figure S11.** Comparison of clustering performed by STRUCTURE and DAPC analysis. **Figure S12.** Discriminant Analysis of Principal Components Loading Plot. **Figure S13.** Comparison of clustering performed by DAPC using the whole (1,215 SNPs) and the linkagedisequilibrium-pruned (326 SNPs) SNPs datasets. **Figure S14.** Sphericity index and the length of pruned values for the selected core collection individuals. (PDF 1.72 MB)

## Abbreviations

AFLP: Amplified fragment Length Polymorphism
BLAST: Basic Local Alignment Search Tool
BIC: Bayesian Information Criterion
CPU: Central Processing Unit
DAPC: Discriminant Analysis of Principal Components
DNA: Deoxyribose Nucleic Acid
FAO: Food and Agriculture Organization of the United Nations
F_IS_: Inbreeding Coefficient
Fst: Fixation Index
GDR: Genome Database for Rosaceae
GnpIS: Genetic and Genomic Information System
GSI: Gametophytic Self-Incompatibility
GUI: Graphical User Interface
H_e_: Expected heterozygosity
H_eq_: Mutation-drift equilibrium
HWE: Hardy Weinberg equilibrium
H_o_: Observed heterozygosity
INRA: French National Institute for Agricultural Research
LD: Linkage Disequilibrium
LG: Linkage Group
MAF: Minor Allele Frequency
Mb: Megabase
MCIA: Mésocentre de Calcul Intensif Aquitain
MCMC: Monte Carlo Markov Chain
NCBI: National Center for Biotechnology Information
NGS: Next Generation Sequencing
PC: Principal Component
PCA: Principal Component Analysis
PCoA: Principal Coordinate Analysis
PGTB: Bordeaux Genome-Transcriptome facility
SNP: Single Nucleotide Polymorphism
SSR: Simple Sequence Repeats
TORQUE: Terascale Open-source Resource and QUEue Manager
TPM: Two-Phase Mutation
UEA: Fruit Tree Experimental Unit
USA: United States of America
