# Supplementary material for: Genetic diversity, linkage disequilibrium, population structure and construction of a core collection of Prunus avium L. landraces and bred cultivars
Source: BMC Plant Biol. 2016 Feb 24;16:49. doi: 10.1186/s12870-016-0712-9 (PMC4765145; doi:10.1186/s12870-016-0712-9)

Figure S1. Workflow of STRUCTURE 2.3.4 software implementation at MCIA cluster nodes. After setting properly the Data & Parameters in the frontend with a GUI, STRUCTURE 2.3.4's user uploads them in the resource handler (TORQUE) and (MAUI) batch scheduler of the MCIA's computing infrastructure. Three Python scripts are used here to distribute, run and collect the results before downloading them back for analysis and display

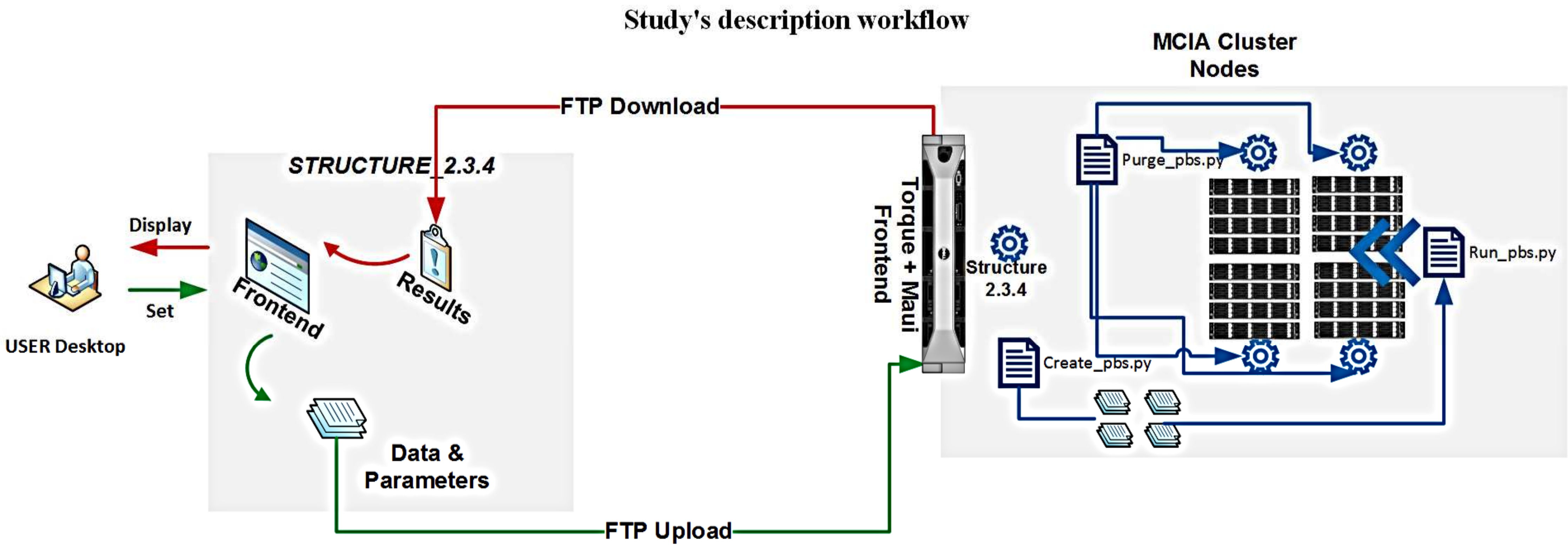

Figure S2. Genome coverage of 1,215 and 326 SNP sets across the eight linkage groups of sweet cherry. Physical position in the peach genome v2.0 (Mb) [27] and the estimated genetic distance in sweet cherry [28] are shown in y-axis. Putative centromere positions in the peach genome are shown by red crosses [27]

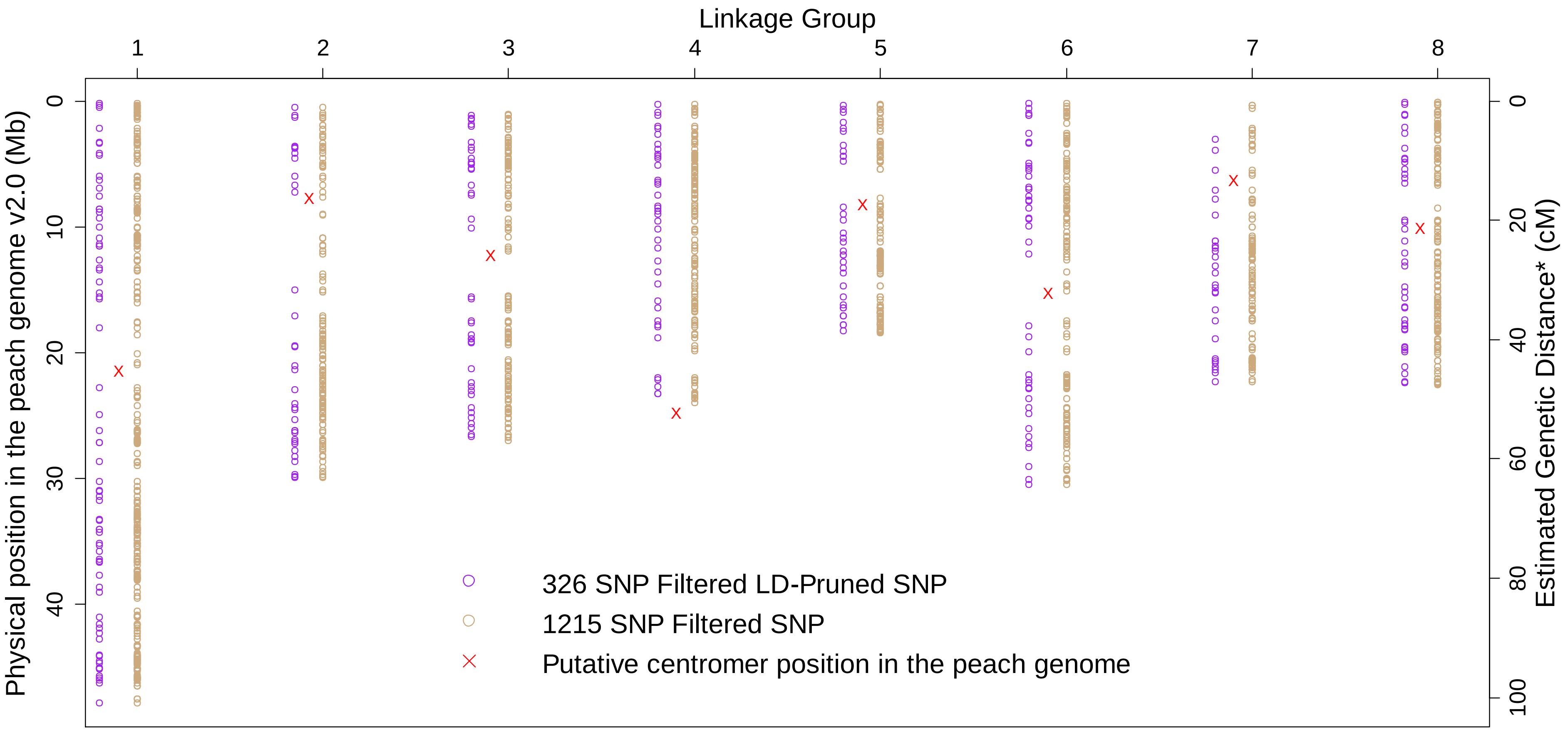

Figure S3 Graphical method (as in Evanno et al., 2005) allowing the detection of the number of groups K using ΔK

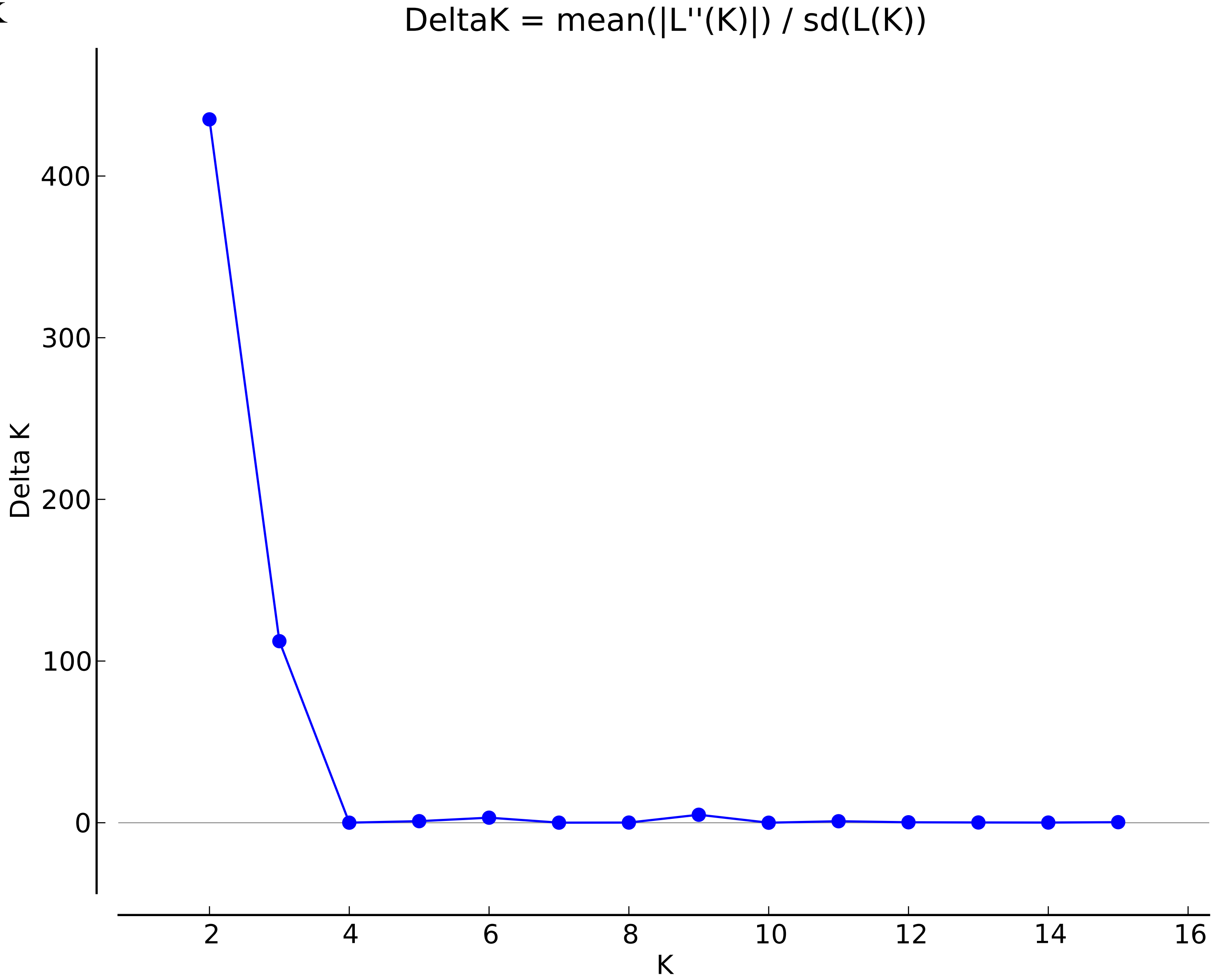

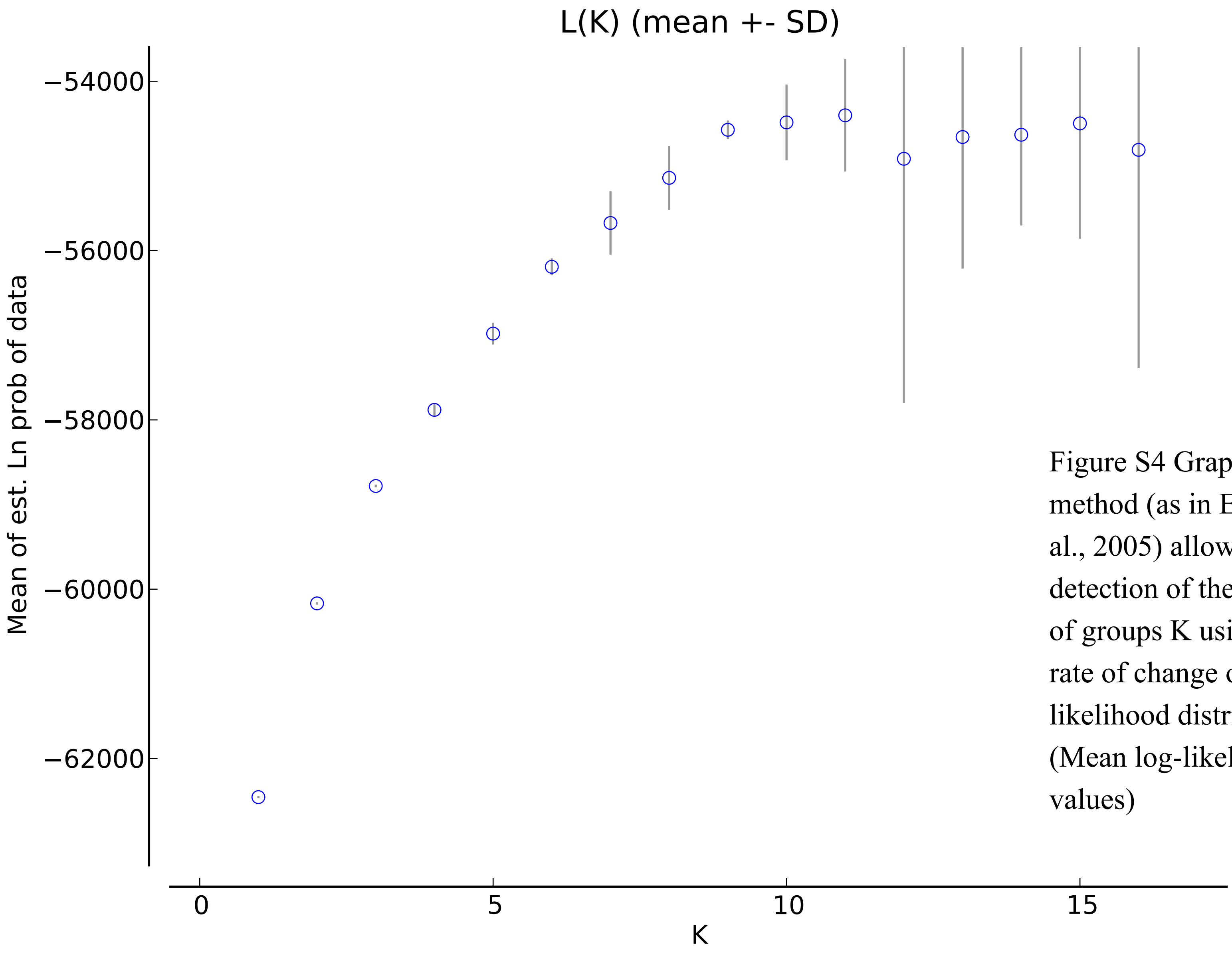

Figure S4 Graphical method (as in Evanno et al., 2005) allowing the detection of the number of groups K using the rate of change of the likelihood distribution (Mean log-likelihood values)

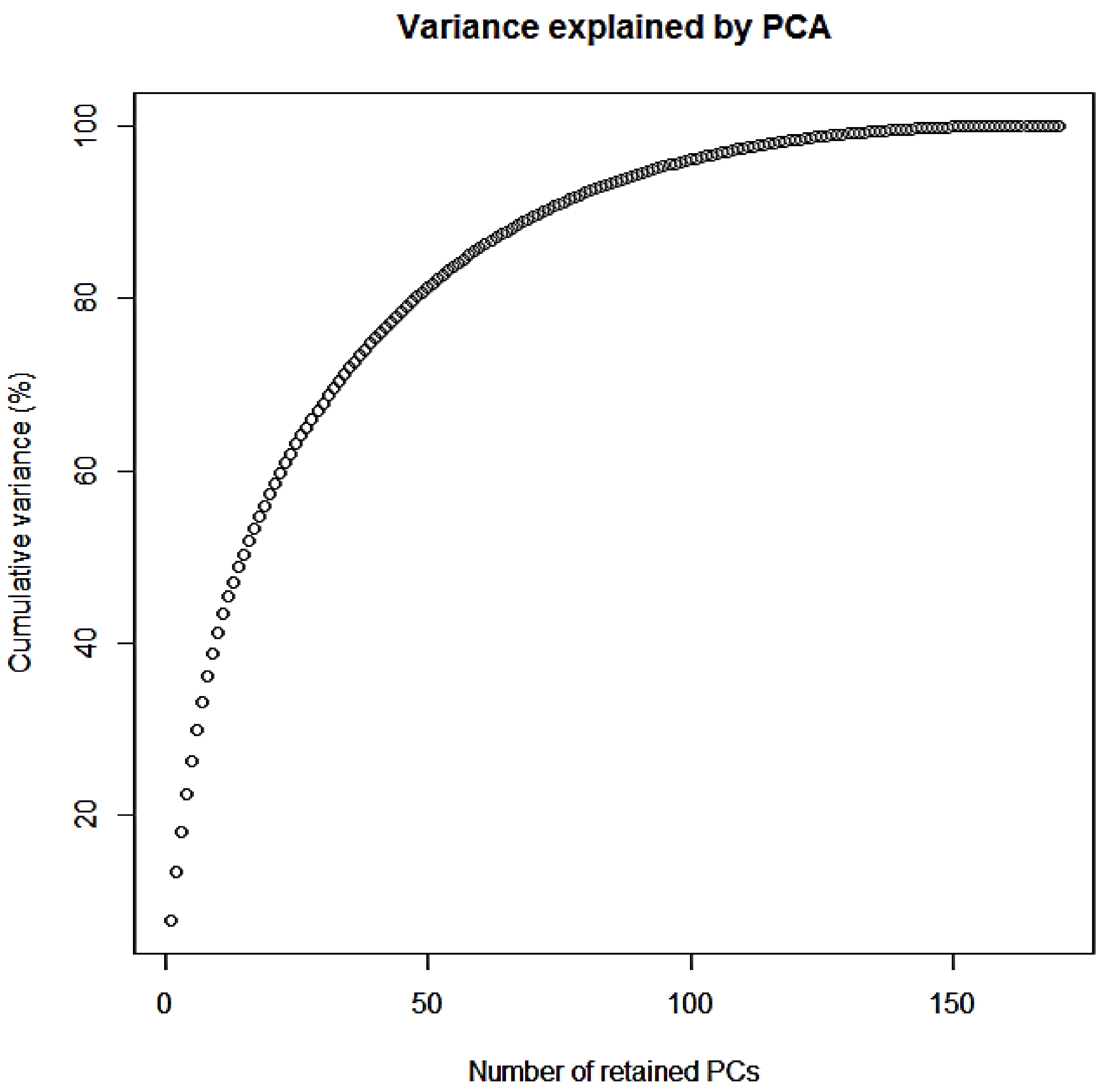

Figure S5. Cumulative variance explained by the principal component analysis (PCA) relative to the number of principal components (PCs) retained in the analysis

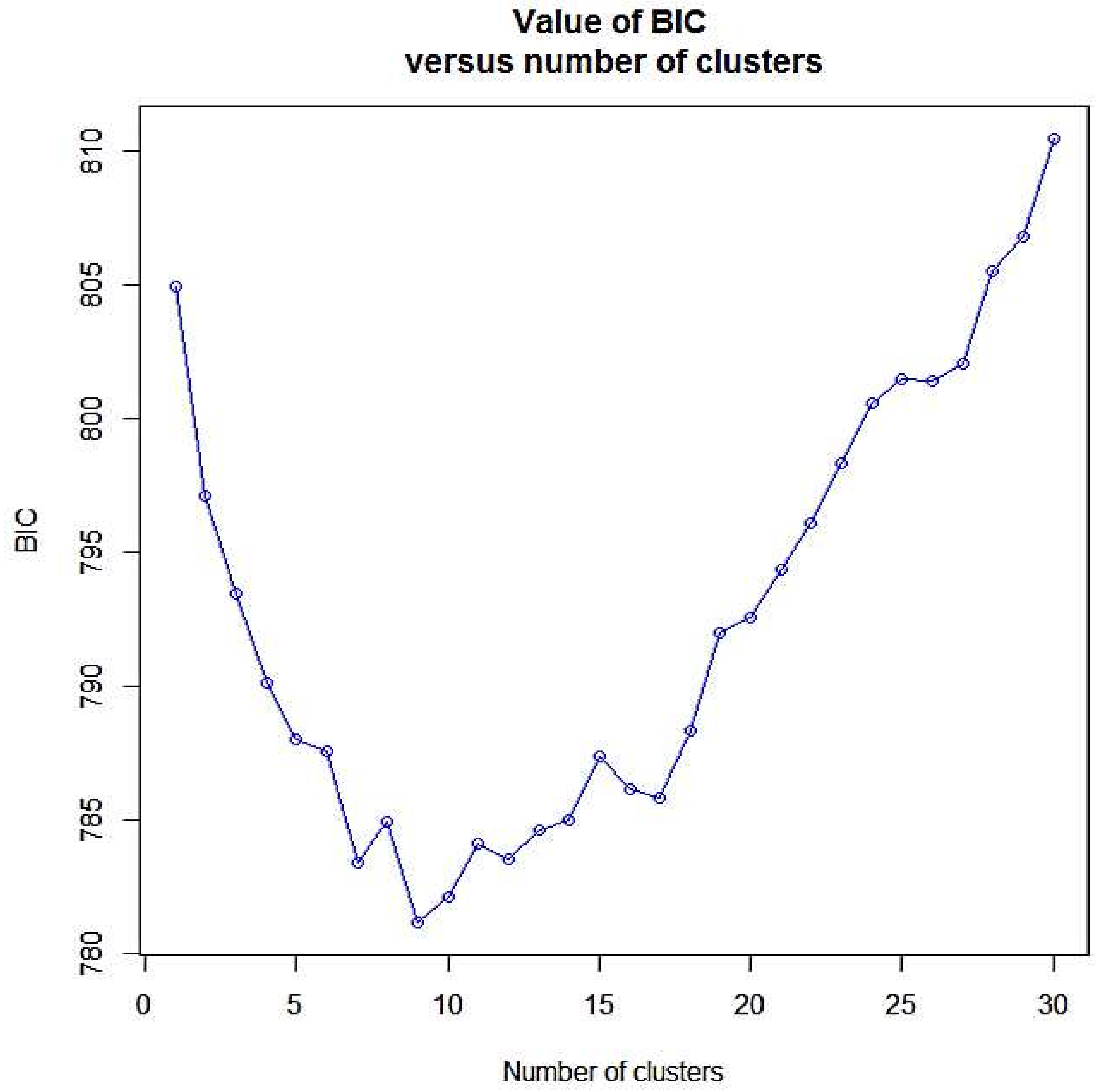

Figure S6. Selection of the optimal number of clusters in the DAPC using the lowest Bayesian Information Criterion (BIC)

Figure S7. Cross-validation procedure to choose the optimal number of Principal Components for the DAPC analysis

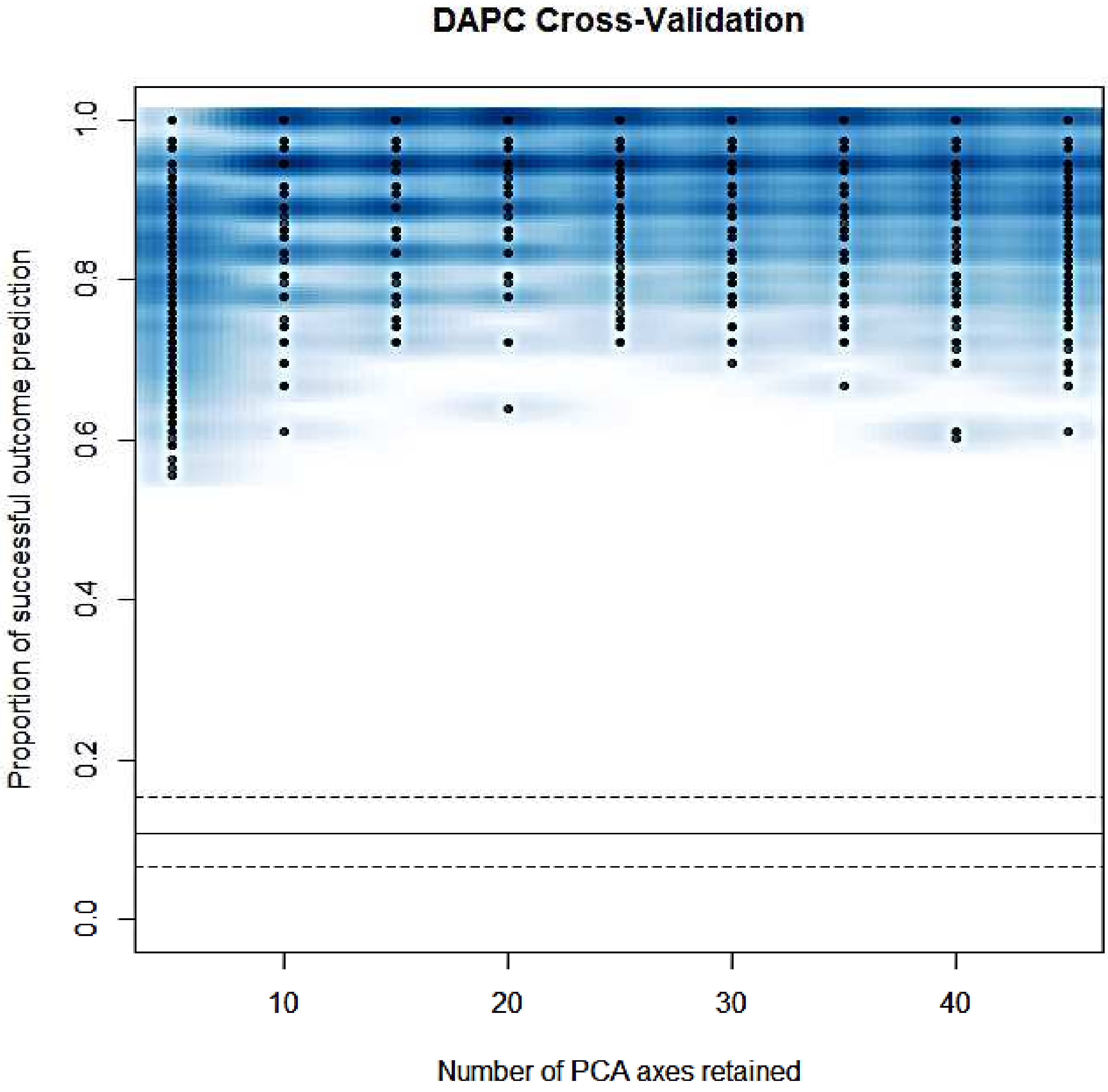

Figure S8. Eigenvalues of retained discriminant functions in the DAPC analysis

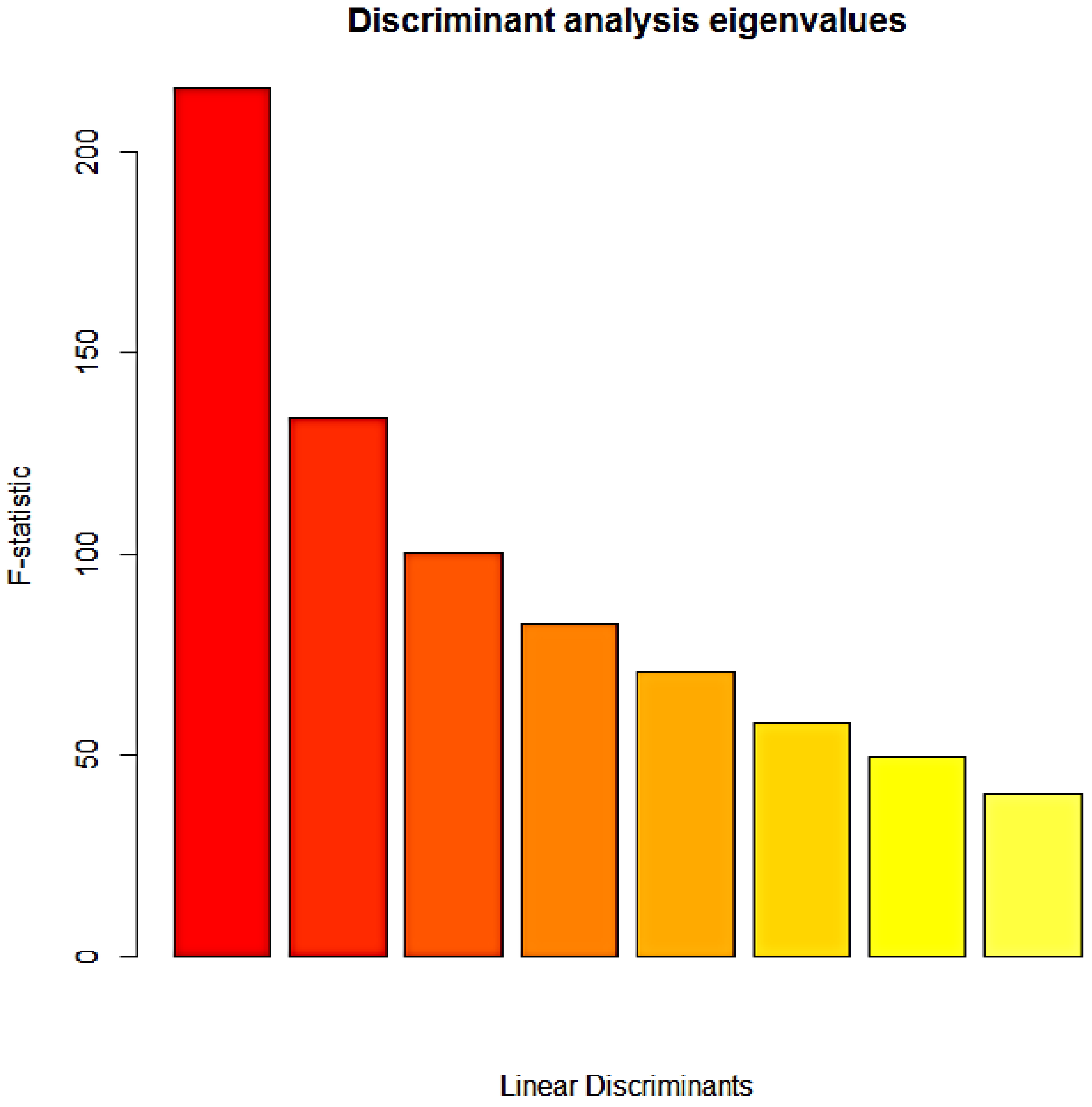

Figure S9. Assignment plots from DAPC for a K of nine populations.
The degree of shading (from white to red) indicates the confidence that each accession is assigned to a particular genetic cluster. Crosses indicate the most confidence cluster (closer color to red). Accession codes are reported in Additional file 1: Table S1

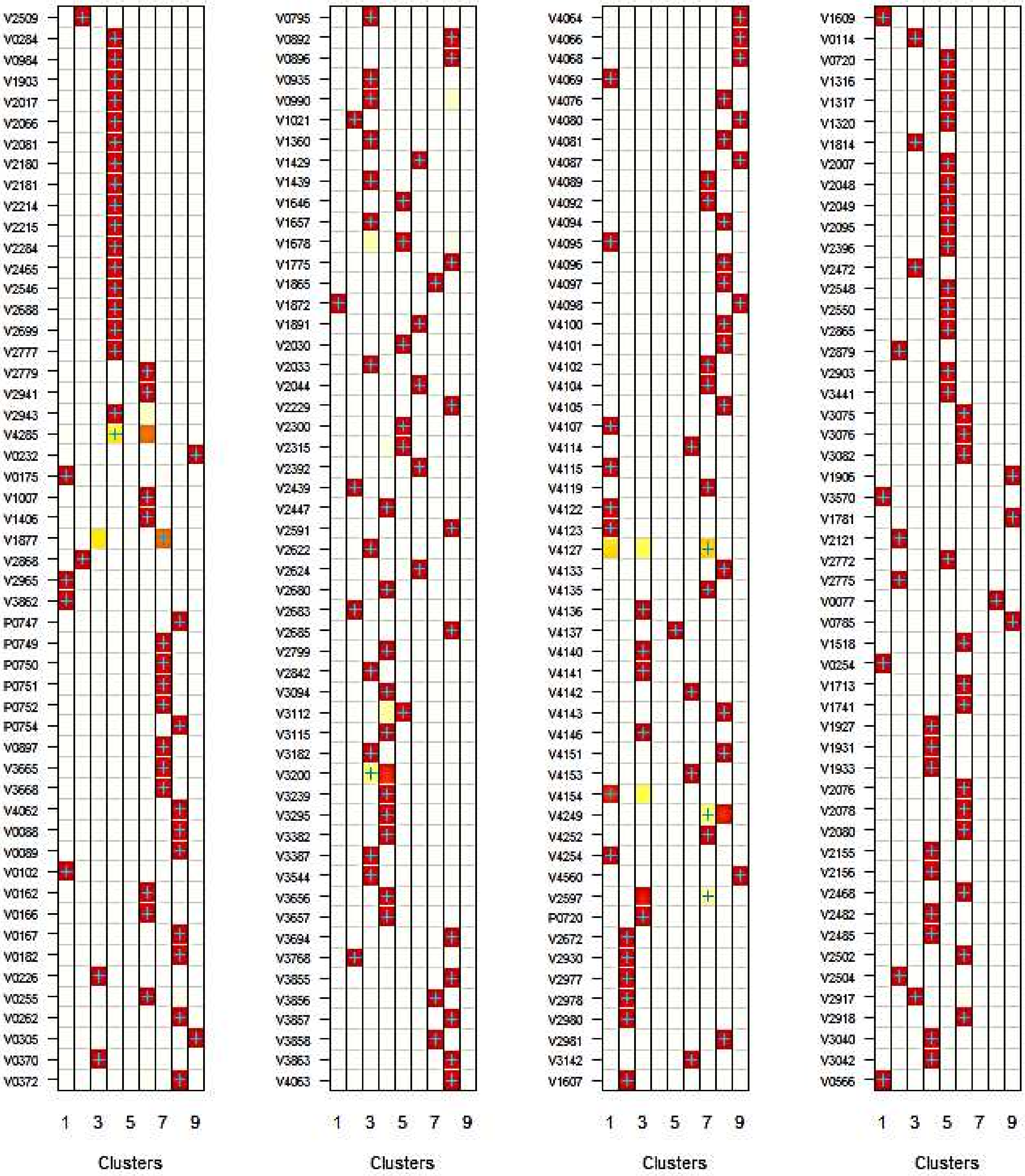

Figure S10. Comparison of clustering performed by DAPC (K=9) and origin of cultivars and landraces. Square size represents the number of individuals in each group

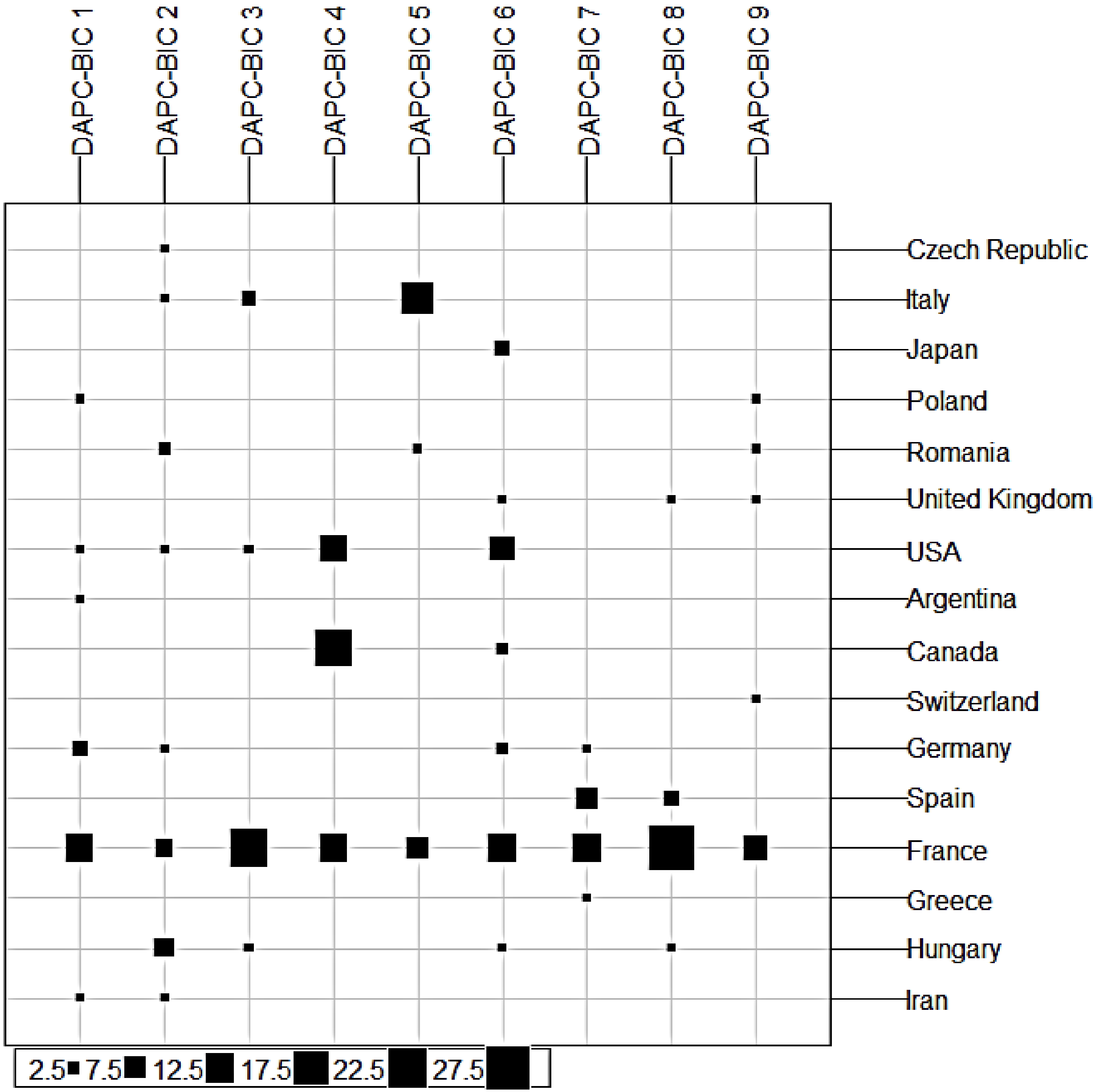

Figure S11. Comparison of clustering performed by STRUCTURE and DAPC analysis. Comparison of STRUCTURE (K=9) and DAPC (K=9) are shown: (a) all admixed individuals are included in their best-hit cluster, (b) all admixed individuals identified in STRUCTURE are included in a different cluster (10th cluster). DAPC groups were randomly called in top and bottom figures by adegenet 2.0 software [31, 32]. Square size represents the number of individuals in each group.

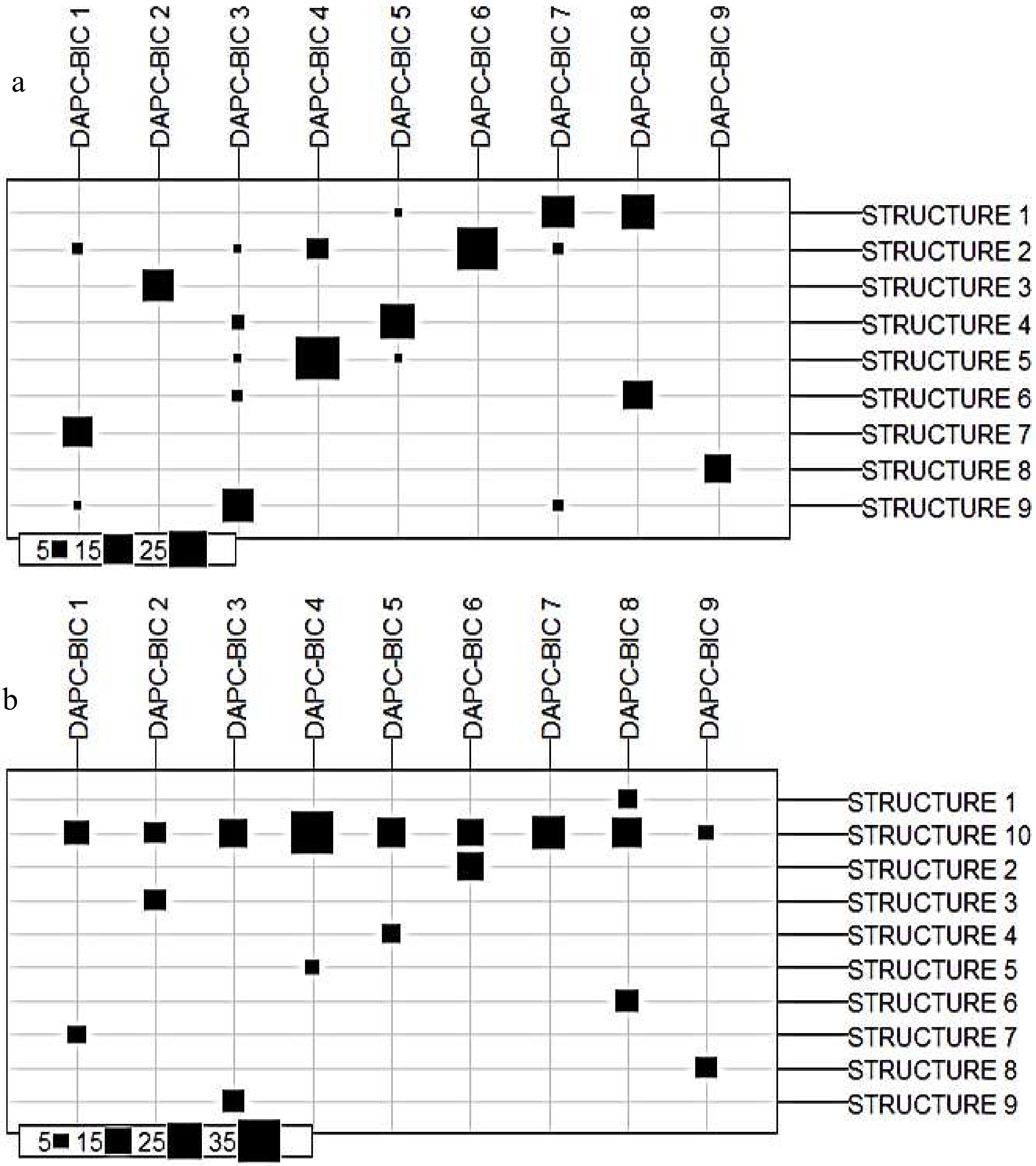

Figure S12. Discriminant Analysis of Principal Components Loading Plot. Plot shows which alleles are best at discriminating among clusters

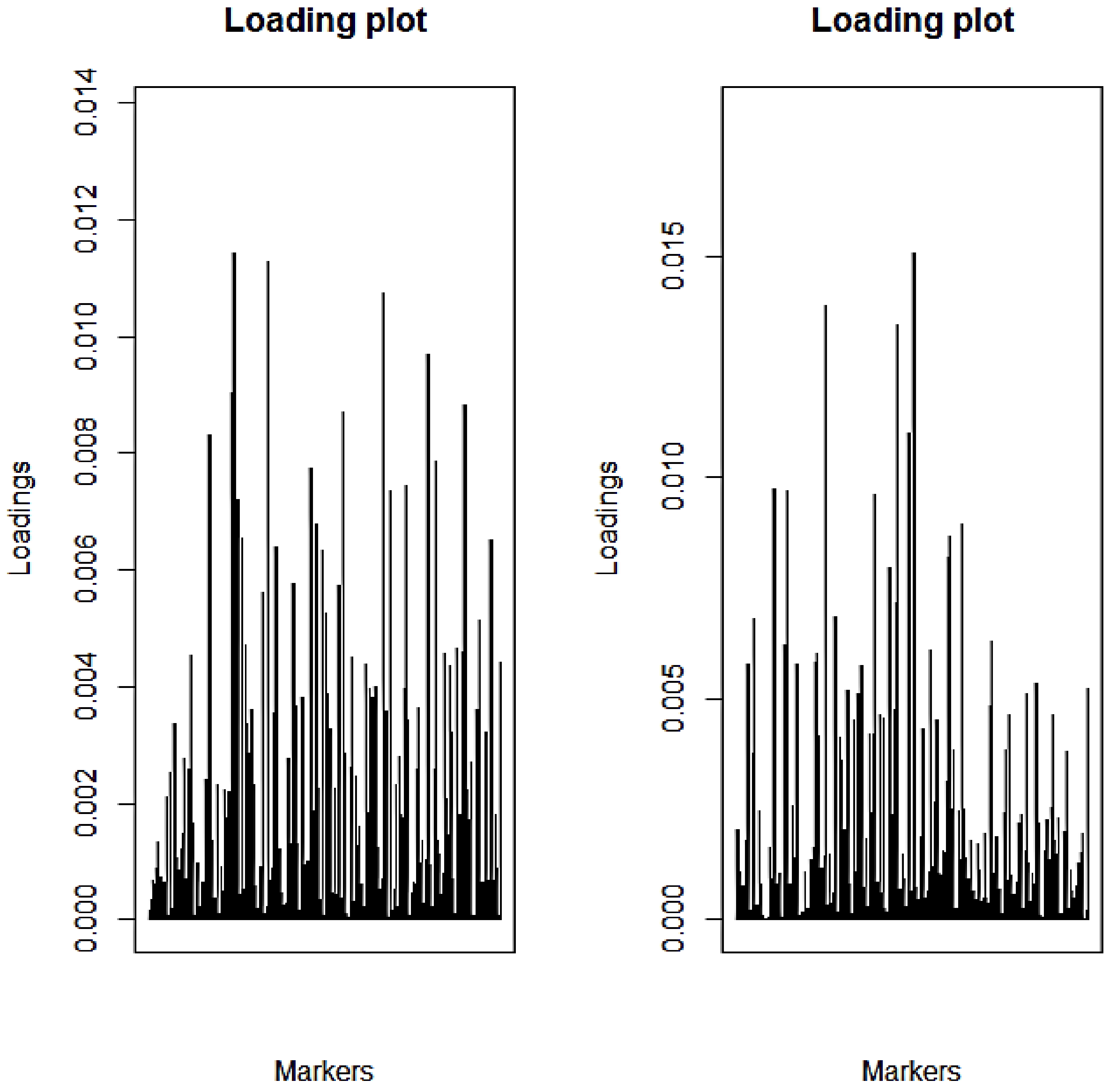

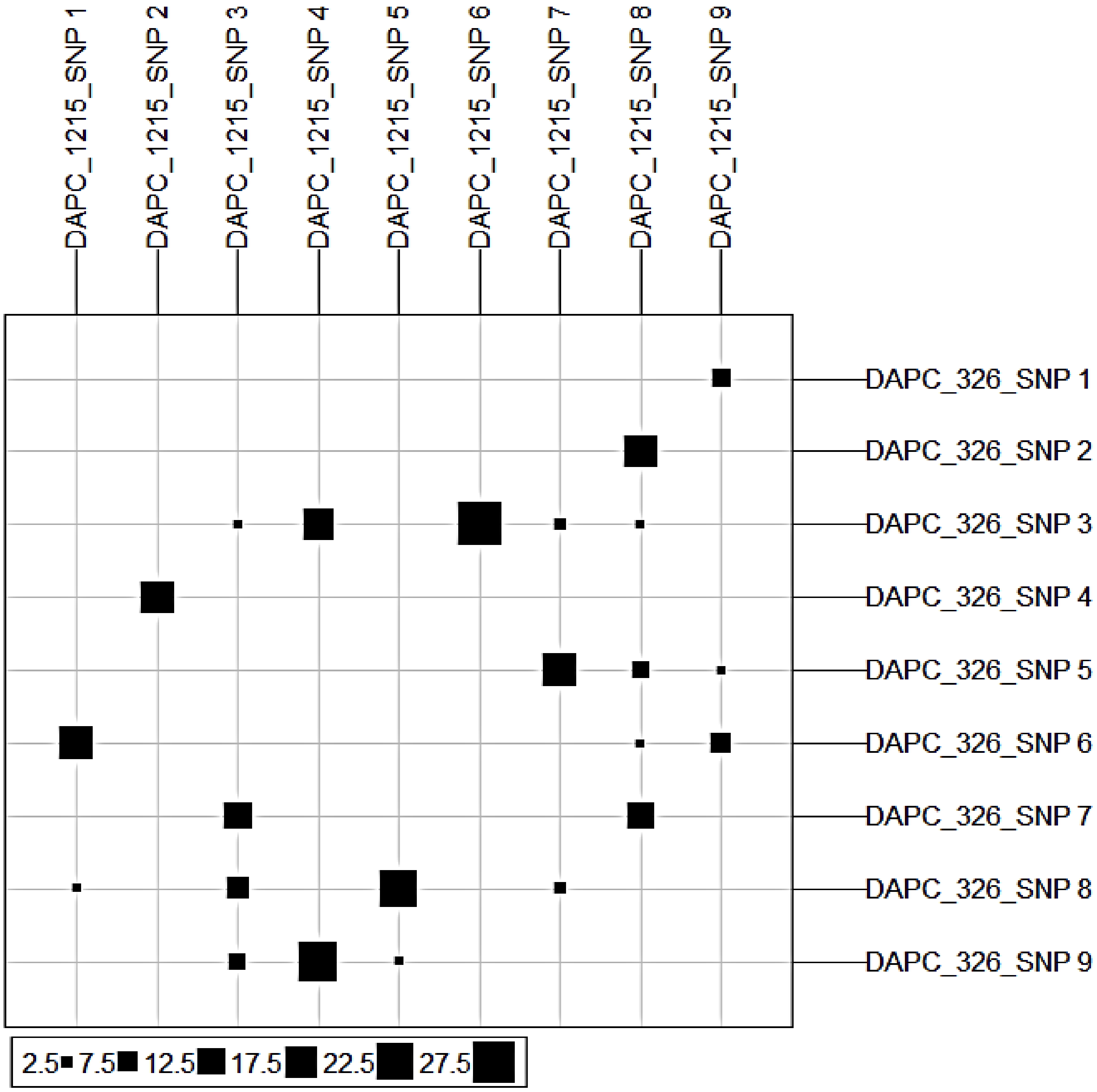

Figure S13. Comparison of clustering performed by DAPC using the whole (1,215 SNPs) and the linkage-disequilibrium-pruned (326 SNPs) SNPs datasets. Square size represents the number of individuals in each group

Figure S14. Sphericity index and the length of pruned values for the selected core collection individuals

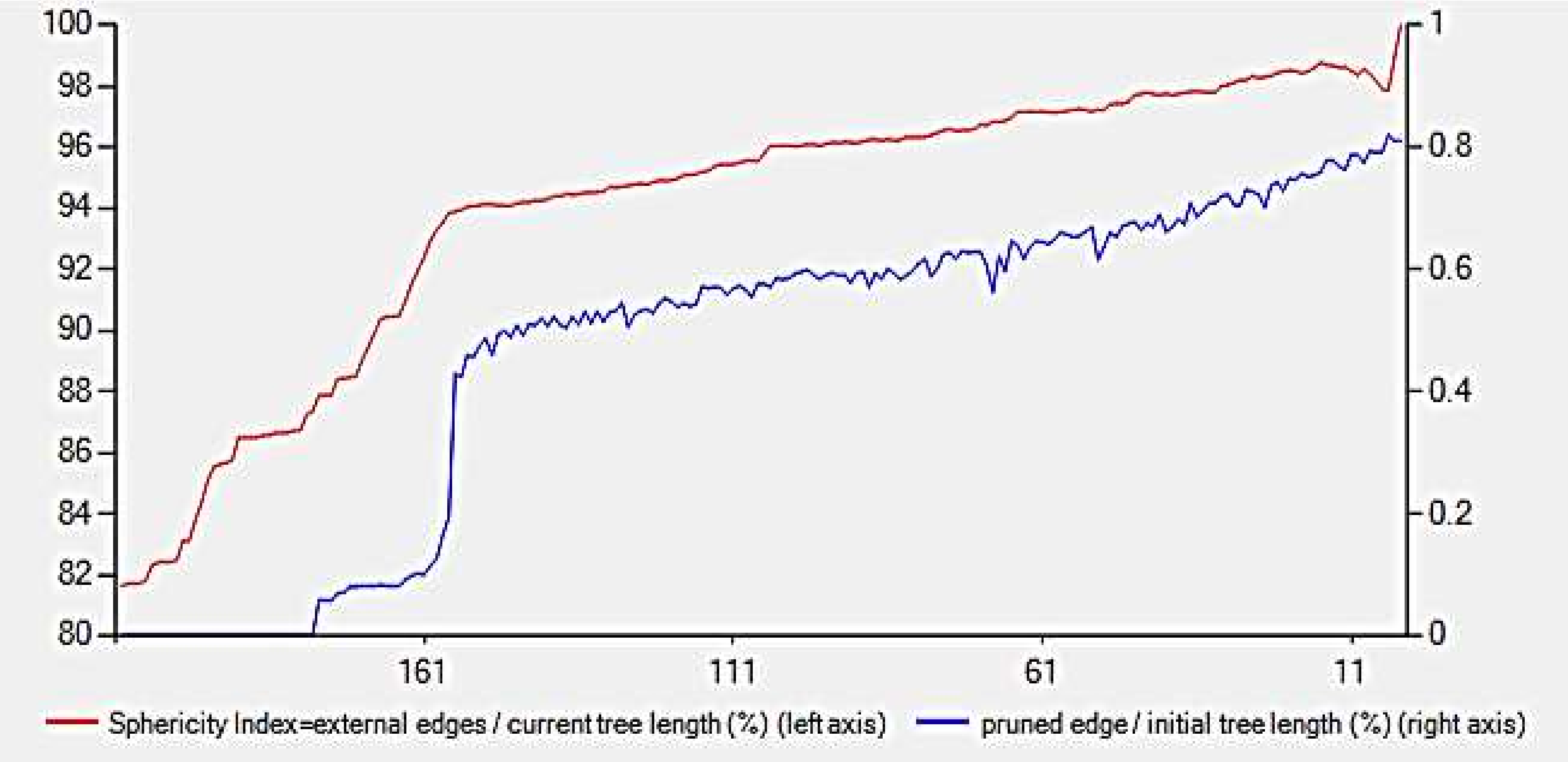

Supplement: Additional file 2: — Figure S1. Workflow of STRUCTURE 2.3.4 software implementation at MCIA cluster nodes. Figure S2. Genome coverage of 1,215 and 326 SNP sets across the eight linkage groups of sweet cherry. Figure S3. Graphical method (as in Evanno et al. 2005) allowing the detection of the number of groups K using ΔK. Figure S4. Graphical method (as in Evanno et al., 2005) allowing the detection of the number of groups K using the rate of change of the likelihood distribution (Mean log-likelihood values). Figure S5. Cumulative variance explained by the principal component analysis (PCA) relative to the number of principal components (PCs) retained in the analysis. Figure S6. Selection of the optimal number of clusters in the DAPC using the lowest Bayesian Information Criterion (BIC). Figure S7. Cross-validation procedure to choose the optimal number of Principal Components for the DAPC analysis. Figure S8. Eigenvalues of retained discriminant functions in the DAPC analysis. Figure S9. Assignment plots from DAPC for a K of nine populations. Figure S10. Comparison of clustering performed by DAPC (K=9) and origin of cultivars and landraces. Figure S11. Comparison of clustering performed by STRUCTURE and DAPC analysis. Figure S12. Discriminant Analysis of Principal Components Loading Plot. Figure S13. Comparison of clustering performed by DAPC using the whole (1,215 SNPs) and the linkagedisequilibrium-pruned (326 SNPs) SNPs datasets. Figure S14. Sphericity index and the length of pruned values for the selected core collection individuals. (PDF 1.72 MB) [file 12870_2016_712_MOESM2_ESM.pdf]
